# Changes in Metabolic Profiles of Human Oral Cells by Benzylidene Ascorbates and Eugenol

**DOI:** 10.3390/medicines5040116

**Published:** 2018-10-31

**Authors:** Hiroshi Sakagami, Masahiro Sugimoto, Yumiko Kanda, Yukio Murakami, Osamu Amano, Junko Saitoh, Atsuko Kochi

**Affiliations:** 1 Meikai University Research Institute of Odontology (M-RIO), 1-1 Keyakidai, Sakado, Saitama 350-0283, Japan; 2 Institute for Advanced Biosciences, Keio University, Tsuruoka 997-0052, Japan; msugi@sfc.keio.ac.jp; 3 Health Promotion and Preemptive Medicine, Research and Development Center for Minimally Invasive Therapies, Tokyo Medical University, Shinjuku, Tokyo 160-0022, Japan; 4 Research and Development Center for Precision Medicine, University of Tsukuba, Tukuba, Ibaraki 305-8550, Japan; 5 Department of Microscope, Meikai University School of Dentistry, 1-1 Keyakidai, Sakado, Saitama 350-0283, Japan; k-yumiko@dent.meikai.ac.jp; 6 Division of Oral Diagnosis and General Dentistry, Meikai University School of Dentistry, 1-1 Keyakidai, Sakado, Saitama 350-0283, Japan; ymura@dent.meikai.ac.jp; 7 Division of Anatomy, Meikai University School of Dentistry, 1-1 Keyakidai, Sakado, Saitama 350-0283, Japan; oamano@dent.meikai.ac.jp; 8 Ichijokai Hospital, 4-26-1 Kitakokubu, Ichikawa, Chiba 272-0836, Japan; junchan@kve.biglobe.ne.jp (J.S.); ichi-1@ya2.so-net.ne.jp (A.K.)

**Keywords:** metabolomics, oral cell, benzaldehyde, eugenol, inflammation, cytotoxicity

## Abstract

**Background:** Sodium-5,6-benzylidene-L-ascorbate (SBA), and its component units, benzaldehyde (BA) and sodium ascorbate (SA), are known to exert antitumor activity, while eugenol exerts anti-inflammatory activity. To narrow down their intracellular targets, metabolomic analysis was performed. **Methods:** Viable cell number was determined by the 3-(4,5-dimethylthiazol-2-yl)-2,5-diphenyltetrazolium bromide (MTT) method. Fine cell structures were observed under transmission electron microscope. Cellular metabolites were extracted with methanol and subjected to capillary electrophoresis-mass spectrometry (CE-MS) for quantification of intracellular metabolites. **Results:** SBA was cleaved into BA and SA under acidic condition. Among these three compounds, BA showed the highest-tumor specificity in vitro against human oral squamous cell carcinoma (OSCC) cell line. BA did not induce the vacuolization in HSC-2 OSCC cells, and its cytotoxicity was not inhibited by catalase, in contrast to SBA and SA. Only BA suppressed the tricarboxylic acid (TCA) cycle at early stage of cytotoxicity induction. Eugenol more rapidly induced the vacuolization and suppressed the TCA cycle in three human normal oral cells (gingival fibroblast, periodontal ligament fibroblast, pulp cell). Neither BA nor eugenol affected the ATP utilization, further supporting that they do not induce apoptosis. **Conclusions:** The present study demonstrated for the first time that both BA and eugenol suppressed the TCA cycle in tumor cells and normal cells, respectively. It is crucial to design methodology that enhances the antitumor potential of BA and reduces the cytotoxicity of eugenol to allow for safe clinical application.

## 1. Introduction

**Figure 1:**
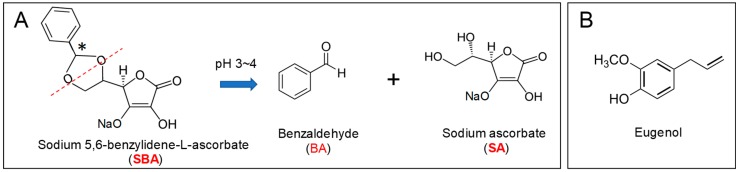
Chemical structure of sodium 5,6-benzylidene-l-ascorbate (SBA) and its hydrolyzed products (benzaldehyde (BA), sodium ascorbate (SA)) (**A**) and eugenol (**B**). SBA has two diastereomers (*S* and *R* configurations at stereogenic center indicated by asterisk in A) present at 31% and 69% (determined by chromato-integrator), respectively [6,7].

Benzaldehyde (BA) is an antitumor principle of the volatile fraction of figs [1]. Several BA derivatives have been prepared for clinical application. Oral administration of β-cyclodextrin benzaldehyde inclusion compound [2] and intravenous administration of 4,6-benzylidene-α-d-glucose [3] or sodium-5,6-benzylidene-l-ascorbate (SBA) [4,5] to patients with advanced, inoperable carcinoma induced remarkable necrotic changes of the tumor, although they showed weak or no antitumor activity against implanted tumors in mice. SBA had no apparent host immunopotentiation activity such as stimulation of cytokine action or production; activation of monocyte or polymorphonuclear cells; or modulation of poly (ADP-ribose) glycohydrolase activity, suggesting the antitumor activity of SBA might be produced by direct action of authentic SBA or its metabolized form(s) [5]. Using a newly established high-performance liquid chromatography (HPLC) separation technique [6], we demonstrated that under acidic condition the acetal linkage in SBA is hydrolyzed, producing benzaldehyde (BA) and sodium ascorbate (SA) [7] (Figure 1A). However, BA level was not changed during incubation for 48 h in culture medium [7].

In vitro study with human oral squamous cell carcinoma (OSCC) cell lines (HSC-2, HSC-3, and HSC-4) and human normal oral cells (gingival fibroblast (HGF), periodontal ligament fibroblast (HPLF), and pulp cell (HPC)) demonstrated that tumor-specificity of BA (TS = 8.8) was four times higher than that of SBA (TS = 2.0), and that neither compounds induced apoptosis (internucleosomal DNA fragmentation, caspase-3, caspase-8, and caspase-9 activation) in OSCC cell line (HSC-2) [8,9], in contrast to HL-60 human promyelocytic leukemic cells [10]. SBA and SA showed common biological properties such as apoptosis induction of HL-60 cells [10], cytotoxicity augmentation with cupper ions, radical generation, and prooxidant action (oxidation potential, hydrogen peroxide production, and methionine oxidation) but showed different properties such as a propensity to react with iron and cysteine analog and catalase sensitivity [11,12,13,14,15] (Table 1). However, to our knowledge, comparative metabolomic study of SBA or SA with BA has not been reported.

In dentistry, zinc oxide-eugenol formulations have been used for many years as bases, liners, cements, and temporary restorative materials [16], and were previously considered as the preferred material for root canal fillings [17]. However, zinc oxide-eugenol released cytotoxic concentrations of eugenol (Figure 1B) [18], and induced chronic inflammation, without healing the pulp, nor forming the dentin bridge up to 12 weeks postoperatively [19]. However, due to the lack of randomized clinical trials, long-term follow-up studies, and proper coronal sealing, whether eugenol is the best root canal filling material for endodontically treated deciduous teeth is still questionable [20,21]. Eugenol induced very rapid and irreversible cell death [22,23] in both human OSCC cell lines (HSC-2, HSC-4, Ca9-22) and normal oral cells (HGF, HPLF, and HCP) to comparable extents [23]. Eugenol induced apoptotic cell death in human promyelocytic leukemia [24], colon cancer [25], and breast cancer cells [26], but not in human normal oral cells and OSCC cell lines [23].

Apoptosis and non-apoptosis are two alternative forms of cell death, with well-defined morphological and biochemical differences [27]. One crucial physiological difference between apoptotic and non-apoptotic cells is the intracellular ATP level. Since apoptosis is an energy-dependent process, a decrease in ATP to below critical levels may impede the execution of apoptosis and promote necrosis [28,29]. This was supported by our findings that sodium fluoride (NaF), which induced apoptosis in HSC-2 cells [30,31], increased the ATP utilization (assessed by AMP/ATP ratio) [32], whereas eugenol, which induced non-apoptosis [19], did not significantly change the ATP utilization in OSCC cells [33]. However, to our knowledge, the effect of eugenol on cellular metabolites of human normal oral cells has not been investigated.

In the present study, we investigated which metabolic pathways are mostly affected by short treatment with SBA-related compounds and eugenol. To accomplish this, we first determined the minimum exposure time required for the irreversible cell death induction, and then performed the metabolomic analysis. The present study demonstrated for the first time that BA shows several properties distinct from those of SBA and SA, and that both BA and eugenol targeted the tricarboxylic acid (TCA) cycle at early stage of cell death induction.

## 2. Materials and Methods

### 2.1. Materials

The following chemicals and materials were obtained from the indicated companies: Dulbecco’s modified Eagle’s medium (DMEM) from Gibco BRL, Grand Island, NY, USA; fetal bovine serum (FBS), benzaldehyde (MW = 106) (purity: >98%), sodium ascorbate (MW = 198), eugenol (MW = 164) (purity: >98%), NaF, D-mannitol, 20% glutaraldehyde solution, dimethylsulfoxide (DMSO) from Wako Pure Chemical, Osaka, Japan; doxorubicin, catalase (EC1.11.1.6, from bovine liver, 41,000 unit/mg protein) from Sigma-Aldrich Inc., St. Louis, MO, USA; mitomycin C from Merck KGaA, Darmstadt, Germany; 5-fluorouracil (5-FU) from Kyowa, Tokyo, Japan; methotrexate from Nacalai Tesque, Inc., Kyoto, Japan; docetaxel from Toronto Research Chemicals, New York, NY, USA; gefitinib from LC Laboratories^®^, PKC Pharmaceuticals, Inc., Woburn, MA, USA; SBA (MW = 286) from ChemiScience, Tokyo, Japan; culture plastic dishes and plates (96-well) from Becton Dickinson Labware, Franklin Lakes, NJ, USA. Eugenol was dissolved in DMSO at 400 mM before use, and diluted with medium. As a control, cells treated with 0.5% DMSO were used.

### 2.2. Cytotoxic Assay

Human OSCC cell line (HSC-2), purchased from Riken Cell Bank (Ibaragi, Japan), and human oral normal cells (HGF, HPLF, and HPC), established from the first premolar tooth extracted from the lower jaw of a 12-year-old girl [34], were cultured in DMEM supplemented with 10% heat-inactivated FBS. Cells were inoculated at 2 × 10^3^ cells per each well of 96-microwell plate. After 48 h, cells were treated with SBA, BA, or SA, as described below. The viable cell number was then determined by MTT method as described previously [8,9,23].

### 2.3. Fine Cell Structure

HSC-2 cells (2 × 10^5^ cells) were inoculated into 8.4-cm (inner diameter) dish. After 48 h, cells were incubated with 0, 1.25, 2.5, 5, or 10 mM SBA, BA, or SA for 3 h, 3 h, or 30 min, respectively. Near confluent HPC (19 population doubling level (PDL)), HPLF (15 PDL), and HGF (20 PDL) cells were incubated with 2 mM eugenol for 0, 20, or 40 min (HPLF, HGF) or 0, 30, or 60 min (HPC). Aliquots of the cells were washed three times with 5 mL of cold phosphate-buffered saline without calcium and magnesium (PBS (−)) and then fixed for 1 h with 2% glutaraldehyde in 0.1 M cacodylate buffer (pH 7.4) at 4 °C. The cells were scraped with a rubber policemen, pelleted by centrifugation, post-fixed for 90 min with 1% osmium tetraoxide-0.1 M cacodylate buffer (pH 7.4), dehydrated, and embedded in Araldite M (CIBA-GEIGY Swiss; NISSHIN EN Co., Ltd., Tokyo, Japan). Thin sections were stained with uranyl acetate and lead citrate and were then observed under a JEM-1210 transmission electron microscope, Japan Electron Optics Laboratory (JEOL, Co., Ltd., Tokyo, Japan) (magnification: ×3000 or ×5000) at an accelerating voltage of 100 kV [8,9,35].

### 2.4. Processing for Metabolomic Analysis

The cells (2~5 × 10^5^) were inoculated on a 8.4-cm (inner diameter) dish and grown to near confluency. After replacing medium with fresh culture medium, cells were treated for each sample. Experiments were performed twice. The following conditions of incubation time and concentrations were used.

Exp. I: HSC-2 cells were treated for 60 min with 0, 0.625, 1.25, 2.5, 5, or 10 mM SBA for 90 min with 0, 1.25, 2.5, 5, 10, or 20 mM BA or for 30 min with 0, 0.625, 1.25, 2.5, 5, or 10 mM SA. Aliquots of the cells were trypsinized for counting the viable cell number with hemocytometer after staining with trypan blue. The cell numbers recovered from the dishes ranged between 2.65~3.03 × 10^6^ cells (SBA), 1.55~1.84 × 10^6^ cells (BA), and 2.75~3.10 × 10^6^ cells (SA) at the time of cell harvest, respectively.

Exp. II: HSC-2 cells were treated for 70 min without (control), or with 4 or 8 mM SBA, or with 8 or 16 mM BA. HGF (19 PDL), HPLF (15 PDL), and HPC (17 PDL) cells were treated with 2 mM eugenol for 0, 20, 40, 60, or 80 min (HGF, HPLF) or for 0, 30, 60, 80, or 100 min (HPC). The cell numbers recovered from the dishes at the time of cell harvest ranged between 3.83~4.22 × 10^6^ cells (HSC-2), 0.195~0.322 × 10^6^ cells (HGF), 0.280~0.495 × 10^6^ cells (HPLF), and 0.640~0.873 × 10^6^ cells (HPC), respectively. 

Aliquots of cells were washed twice with 10 mL of ice-cold 5% d-mannitol and then immersed for 10 min with 1 mL methanol containing internal standard (25 μmol/L each of methionine sulfone, 2-[*N*-morpholino]-ethanesulfonic acid and d-camphor-10-sulfonic acid). The supernatant (methanol extract) was collected. To 400 µL of the dissolved samples, 400 µL of chloroform and 200 µL of Milli-Q water were added and the mixture was centrifuged at 10,000× *g* for 3 min at 4 °C. The aqueous layer was filtered to remove large molecules by centrifugation through a 5-kDa cut-off filter (Millipore, Billerica, MA) at 9100× *g* for 2.0 h at 4 °C. Three hundred and twenty microliters of the filtrate was concentrated by freeze drying and dissolved in 50 µL of Milli-Q water containing reference compounds (200 µM each of 3-aminopyrrolidine and trimesate) immediately before capillary electrophoresis (CE)-time-of-flight (TOF)-mass spectrometry (MS) analysis [33,35,36].

### 2.5. CE-MS Analysis

The instrumentation and measurement conditions used for CE-TOF-MS were described previously [37,38] with slight modification [36]. 

For cationic metabolite analysis using CE-TOF-MS, a sample was prepared in fused silica capillaries filled with 1 mol/L formic acid as the reference electrolyte [32]. The capillary was flushed with formic acid. Sample solutions (3 nL) were injected at 50 mbar for 5 s and a voltage of 30 kV was applied. The capillary temperature was maintained at 20 °C and the temperature of the sample tray was kept below 5 °C. The sheath liquid was delivered at 10 μL/min. Electrospray ionization (ESI)-TOF-MS was conducted in the positive ion mode. The capillary voltage was set at 4 kV and the flow rate of nitrogen gas (heater temperature = 300 °C) was set at 7 psig. In TOF-MS, the fragmentor, skimmer, and OCT RF voltages were 75, 50, and 125 V, respectively. Automatic recalibration of each acquired spectrum was performed using reference standards. Mass spectra were acquired at a rate of 1.5 cycles/s over a *m*/*z* range of 50–1000.

For anionic metabolite analysis using CE-TOF-MS, a commercially available COSMO (+) capillary, chemically coated with a cationic polymer, was used for separation. Ammonium acetate solution (50 mmol/L; pH 8.5) was used as the electrolyte for separation. Before the first use, the new capillary was flushed successively with the running electrolyte (pH 8.5), 50 mmol/L acetic acid (pH 3.4), and then the electrolyte again for 10 min each. Before each injection, the capillary was equilibrated for 2 min by flushing with 50 mM acetic acid (pH 3.4) and then with the running electrolyte for 5 min [32]. A sample solution (30 nL) was injected at 50 mbar for 30 s, and a voltage of −30 kV was applied. The capillary temperature was maintained at 20 °C and the sample tray was cooled below 5 °C. An Agilent 1100 series pump equipped with a 1:100 splitter was used to deliver 10 μL/min of 5 mM ammonium acetate in 50% (*v*/*v*) methanol/water, containing 0.1 μM Hexakis, to the CE interface. Here, it was used as a sheath liquid surrounding the CE capillary to provide a stable electrical connection between the tip of the capillary and the grounded electrospray needle. ESI-TOF-MS was conducted in the negative ionization mode at a capillary voltage of 3.5 kV. For TOF-MS, the fragmentor, skimmer, and OCT RF voltages were set at 100, 50, and 200 V, respectively. The flow rate of the drying nitrogen gas (heater temperature = 300 °C) was maintained at 7 psig. Automatic recalibration of each acquired spectrum was performed using reference standards ([13C isotopic ion of deprotonated acetic acid dimer (2 CH3COOH–H)]^−^, *m*/*z* 120.03841), and ([Hexakis + deprotonated acetic acid (M + CH3COOH–H)]^−^, *m/z* 680.03554). Exact mass data were acquired at a rate of 1.5 spectra/s over a *m/z* range of 50–1000.

Cation analysis was performed using an Agilent CE capillary electrophoresis system, an Agilent G6220A LC/MSD TOF system, an Agilent 1100 series isocratic HPLC pump, a G1603A Agilent CEMS adapter kit, and a G1607A Agilent CE-ESI-MS sprayer kit [35]. Anion analysis was performed using an Agilent CE capillary electrophoresis system, an Agilent G6220A LC/MSD TOF system, an Agilent 1200 series isocratic HPLC pump, a G1603A Agilent CE-MS adapter kit, and a G7100A Agilent CE-electrospray ionization (ESI) source-MS sprayer kit [35].

### 2.6. Statistical Analysis

Data are expressed as the mean ± standard deviation (S.D.). Raw data of metabolomics analysis were analyzed using our proprietary software, MasterHands [39,40]. Concentrations were calculated using external standards based on relative area (i.e., the area divided by the area of the internal standards). Overall metabolomic profiles were accessed by principal component (PC) analysis (PCA). XLstat (Ver. 2014.1.04, Addinsoft, Paris, France) GraphPad Prism (Version 5.04, GraphPad Software, San Diego, CA, USA), and MeV (Version 4.9.0, http://mev.tm4.org/, Center for Cancer Computational Biology, Dana-Farber Cancer Institute, Boston, MA, USA) were used for PCA and other statistical tests. Differences were considered significant at *p* < 0.05. 

## 3. Results

### 3.1. Distinct Biological Properties of BA from SBA and SA

#### 3.1.1. Catalase Sensitivity

**Figure 2:**
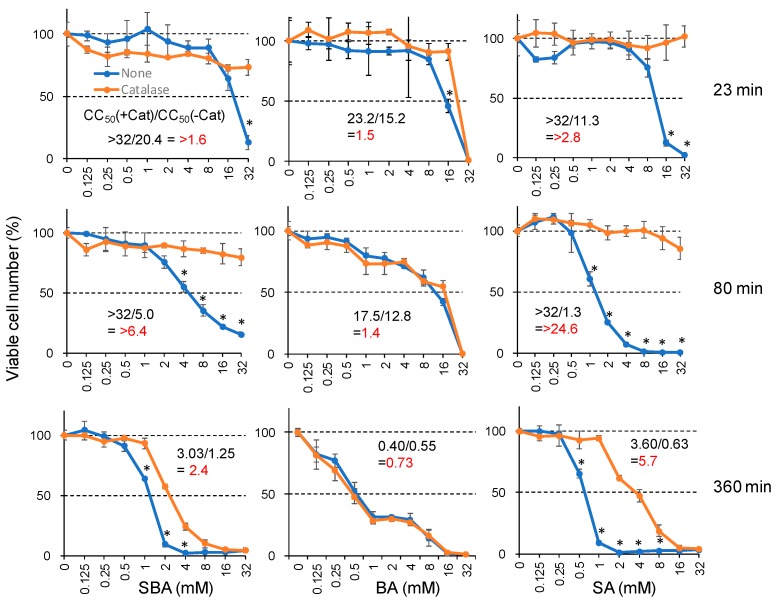
Involvement of extracellularly released hydrogen peroxide in the cytotoxicity induced by SA and SBA, but not BA. HSC-2 cells were incubated for 23, 80, or 360 min with the indicated concentrations of each compound in the presence or absence of catalase (3000 units/mL), and then replaced with fresh medium and incubated for a total of 48 h. Viable cell number was determined by MTT method, and expressed as % of control (drug-free, catalase-free). Each value represents mean ± S.D. of triplicate assays. * Significant difference from control *p* < 0.05.

The addition of catalase (3000 unit/mL) reduced the cytotoxicity of SBA and SA up to >6.4-fold and >24.6-fold, respectively, confirming our previous finding [13]. On the other hand, the cytotoxicity of BA was not affected by the addition of catalase (Figure 2). This suggests that extracellularly released hydrogen peroxide may not be involved in BA-induced cytotoxicity. 

#### 3.1.2. Minimum Exposure Time Required for Irreversible Cell Death Induction

**Figure 3:**
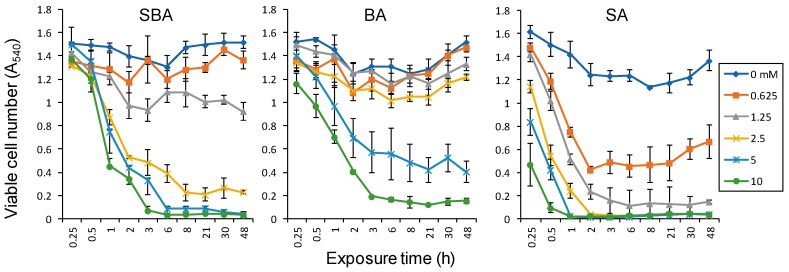
Minimum exposure time required for cytotoxicity induction by SBA, BA, and SA. HSC-2 cells were incubated for the indicated times without (control) or with 0, 0.625, 1.25, 2.5, 5, or 10 mM for each compound, and replaced with fresh drug-free medium. Cells were incubated for a total of 48 h and viable cell number was determined by MTT methods. Each value represents the mean ± S.D. of triplicate assays.

In order to detect the initial event that leads to irreversible cell death, we first determined the minimum exposure time. HSC-2 cells were incubated for various times, and then medium was replaced with drug-free medium and cells were incubated for up to 48 h. Viable cell number was reduced dose- and time-dependently after treatment with any drugs tested. Cytotoxic action of SA was the fastest, reaching the plateau phase of viable cell number reduction after only 2 h. Cytotoxic action of SBA was slightly slower, requiring 3~6 h to reach the bottom. Cytotoxic action of BA was much slower, requiring 8 h to reach the bottom (Figure 3).

#### 3.1.3. Induction of Mitochondrial Vacuolization

**Figure 4:**
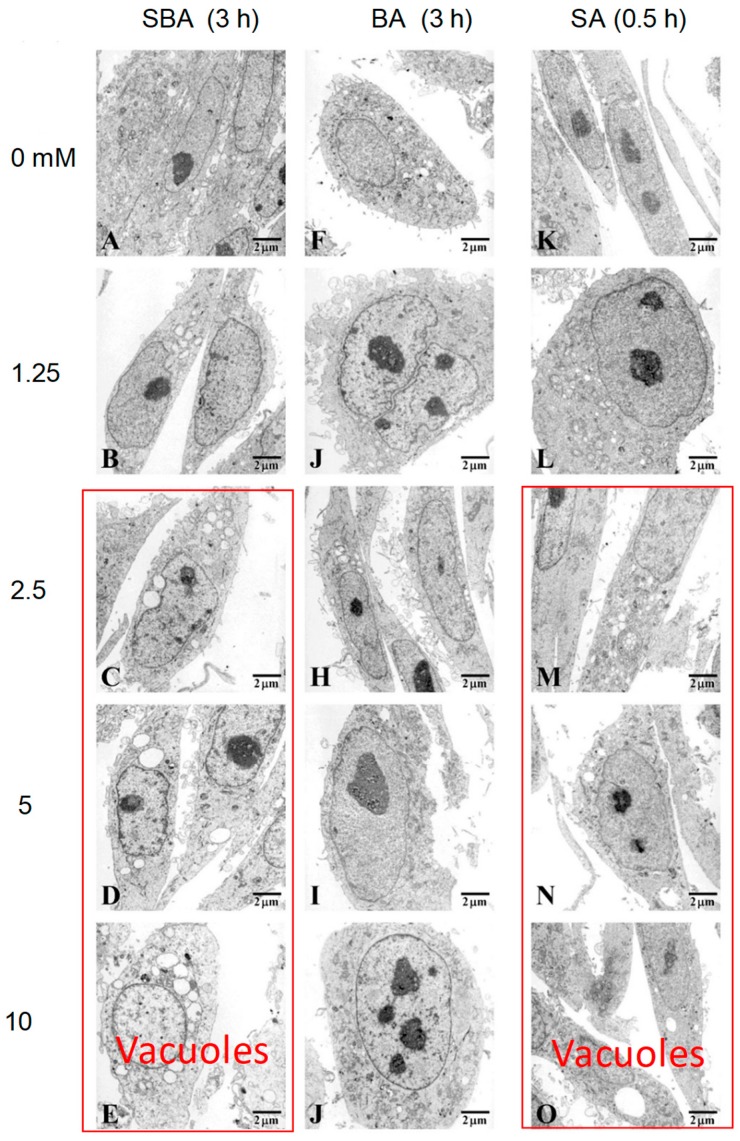
TEM analysis of fine cell structure of HSC-2 cells treated by the indicated concentrations of SBA (**A**–**E**), benzaldehyde (**F**–**J**), and sodium ascorbate (**K**–**O**). Images magnified at ×5000.

When HSC-2 cells were treated for 3 h with SBA and SA at 2.5 mM or higher concentrations, mitochondrial vacuolization and multivesicular bodies appeared (indicated by red box). On the other hand, BA did not induce such morphological changes (Figure 4). 

#### 3.1.4. Effect on TCA Cycle Metabolites

**Figure 5:**
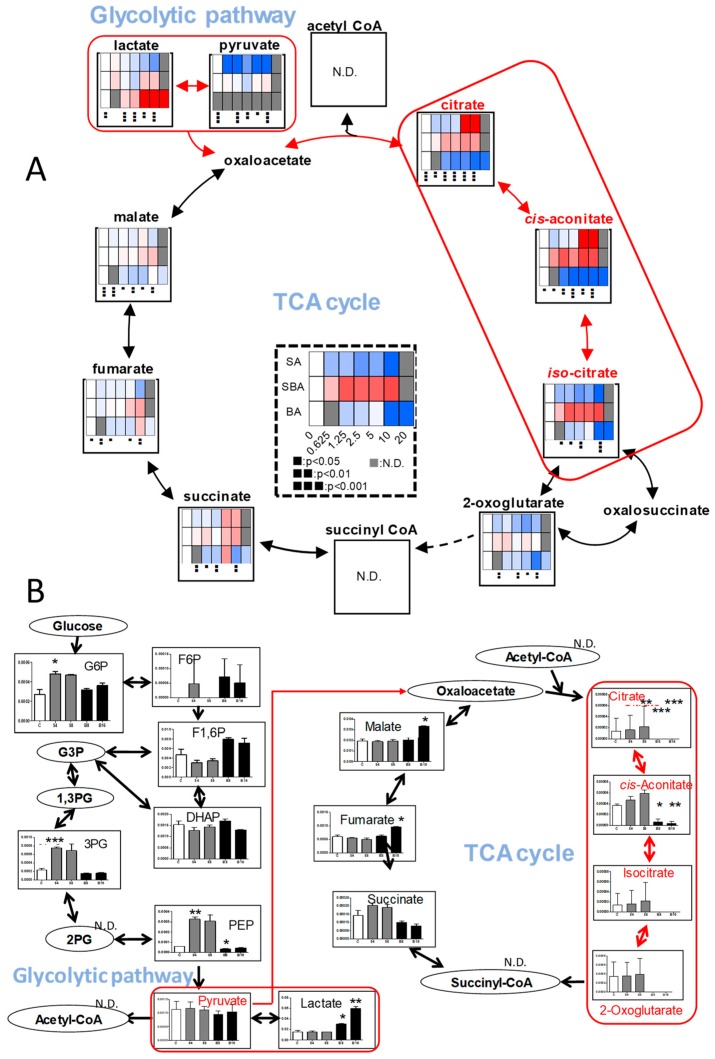
BA, but not SAB nor SA, inhibits the tricarboxylic acid (TCA) cycle. (**A**). Dose-response of SBA (60 min), BA (90 min), SA (30 min). HSC-2 cells were treated as Exp. I in Materials and Methods. Averaged values of quantified metabolites (amol/cell) were calculated by triplicate. To visualize the data in heat maps, each of the values were divided by those of 0 mM, i.e. fold changes were visualized and colored. Blue, white, and red were assigned for the fold changes of 0, 1, and 2, respectively. For the treatments of 0.626 mM, Student’s *t*-tests were used, and for those of 20 mM, no statistical test was conducted. Not detected (N.D.) was colored in gray. One-way ANOVAs were conducted and black boxes were assigned below each heat map. (**B**). Dose-response of SBA and BA. HSC-2 cells were treated as Exp. II in Materials and Methods..Horizontal axis from left to right: C, S4, S8, B8, and B16 represent control, 4 and 8 mM SBA, 8 and 16 mM BA, respectively. Treatment time: 70 min. Control and each group were analyzed using Welch’s test (both tail) and *p*-values were adjusted by Bonferroni correction. * *p* < 0.05, ** *p* < 0.01, and *** *p* < 0.001. Error bar indicates standard deviation of triplicates.

In order to detect early metabolic changes, HSC-2 cells were incubated for only 60, 90, and 30 min with increasing concentrations of SBA, BA, or SA, respectively, and subjected to metabolomic analysis. We found dramatic changes in the TCA cycle after treatment of BA (Figure 5). 

Within 90 min exposure to BA, intracellular concentrations of citrate, *cis*-aconitate, and *iso*-citrate declined to base-line level in HSC-2 cells, whereas that of succinate, fumarate, and malate maintained almost constant levels, indicating the rapid suppression of TCA cycle progression. In contrast, we could not detect such dramatic changes in other pathways (including pyrimidine metabolism, purine metabolism, glycolysis, pentose phosphate pathway, arginine, proline metabolism and urea cycle, glycine, serine and threonine metabolism, alanine, asparagine and aspartate metabolism, lysine metabolism, beta-alanine biosynthesis and metabolism, methionine and cysteine metabolism, histidine metabolism, tryptophan metabolism, phenylalanine, tyrosine metabolism, valine, leucine and isoleucine metabolism, γ-aminobutyric acid (GABA) biosynthesis and metabolism and glutamine metabolism) (Figure S1, Table S1). We checked the reproducibility of the finding (Table S2). The repeated experiment again showed that citrate, *cis*-aconitate, isocitrate, and 2-oxoglutarate were depleted by 8 or 16 mM BA (Figure 5B). 

**Figure 6:**
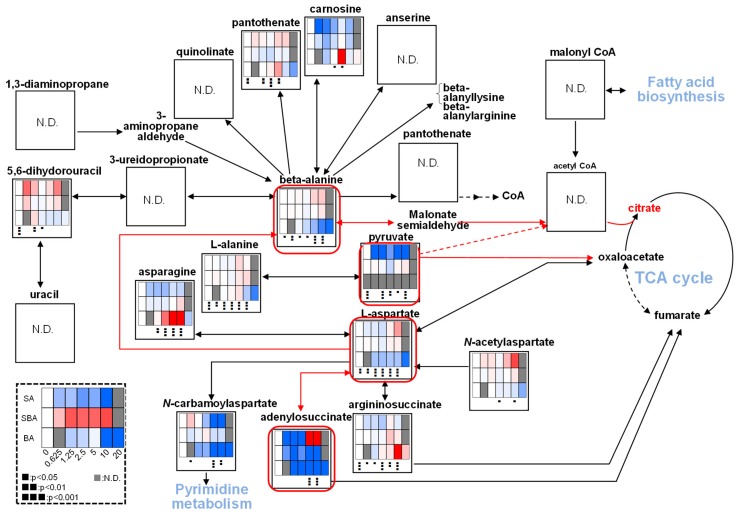
Possible pathway that reduces the supply of citrate by BA. SBA (60 min), BA (90 min), SA (30 min). The procedures used to produce heat maps and statistical analyses were described in Figure 5A.

We next investigated which pathway may be involved in suppressing the TCA cycle. We found that BA increased lactate production, maintaining nearly constant levels of pyruvic acid, suggesting that BA may have reduced the amount of pyruvate that enters the TCA cycle (Figure 5A). We confirmed this finding by repeating the experiment (Figure 5B). BA reduced β-alanine, L-aspartic acid, and adenylosuccinate (Figure 6), which may directly or indirectly reduce the production of citrate.

**Figure 7:**
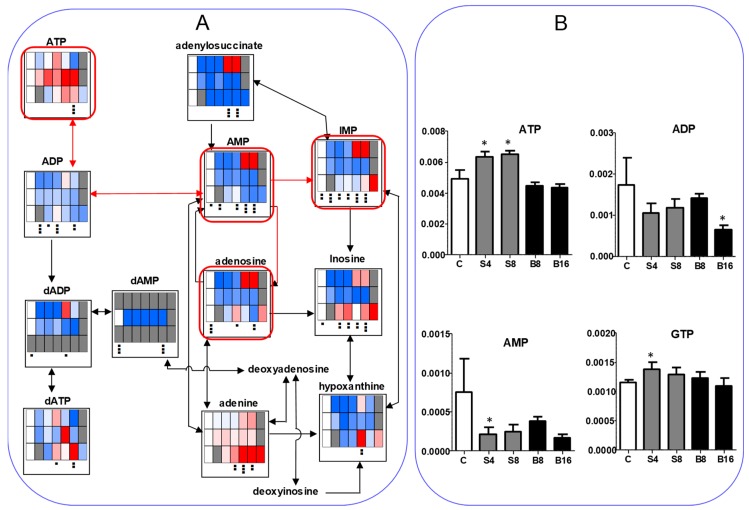
BA as well as SBA, but not, SA, reduce the ATP utilization. (**A**). SBA (60 min), BA (90 min), SA (30 min). HSC-2 cells were treated as Exp. I in Materials and Methods. (**B**). Horizontal axis from left to right: C, S4, S8, B8, and B16 represent control, 4 and 8 mM SBA, 8 and 16 mM BA, respectively. HSC-2 cells were treated as Exp. II in Materials and Methods. Vertical axis, μmol/cell. Each value represents mean ± S.D. (*n* = 3). The procedures used to produce heat maps and statistical analyses were described in the legend of Figure 5. * *p* < 0.05.

We next investigated the effect on ATP utilization (Figure 7). BA treatment reproducibly reduced the AMP to less than half of the control level while it maintained a nearly constant ATP level, indicating the reduction of ATP utilization (Figure 7A). SBA treatment slightly elevated ATP but reduced AMP to one-third of the control level, again indicating the reduction of ATP utilization. On the other hand, SA increased the AMP at higher concentration (5 and 10 mM), suggesting the enhancement of ATP utilization (Figure 7A). We checked the reproducibility that both SAB and BA reduced the AMP utilization (Figure 7B). These data suggest that BA may not induce apoptosis in HSC-2 cells, in accord with our previous report [4,5]. 

We also investigated the changes in redox and amino acids (Figure S1). SBA, BA, and SA increased methionine sulfoxide and oxidized glutathione (GSSG) but reduced Glutathione (GSH) only slightly at higher concentration, suggesting that these compounds showed some minor prooxidant action. It was unexpected that BA dose-dependently reduced GABA, while glutamate was not changed significantly. However, the biological significance of this finding is unclear at present. Glutamine level was kept constant except for at the highest concentration of BA, negating the possibility that BA-induced cell death is not mediated by the deletion of glutamine, one of the energy sources for cell survival.

**Figure 8:**
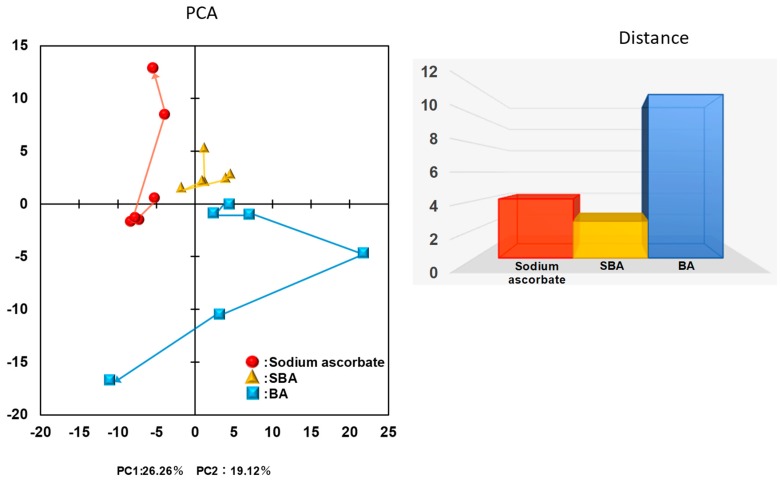
Principal component analysis (PCA) analysis of closeness between SBA related compounds.

Overall metabolomic profiles were accessed by principal component (PC) analysis (PCA) (Figure 8). Plots of SA, SBA, and BA were separated into non-overlapped clusters with each other. Along with the first PC, SBA was clustered at slightly higher values (−2~5) than SA (−8~−4), and BA was clustered at much higher values (2~23) with the exception of two plots. Along with the second PC, SA (−2~13) and SBA (2~6) distributed into an overlapped region, whereas BA distributed at much lower values (0~−17). This indicates that the changes in the intracellular metabolites induced by SBA are more close to those induced by SA, as compared with those induced by BA.

### 3.2. Changes of Metabolic Profiles in Normal Oral Cells Induced by Eugenol

#### 3.2.1. Eugenol Induced Rapid Collapse in Mitochondria

We have previously reported that four dental compounds, hydroquinone, benzoquinone, eugenol, and phtharal, induced irreversible cell death in human oral squamous cell carcinoma (OSCC) cell lines (HSC-2, HSC-4, Ca9-22) and normal human oral cells (HGF, HPLF, HPC) and skin keratinocytes within 4 h, without induction of apoptotic markers. The 50% cytotoxic concentration (CC_50_) of eugenol was about 0.70~0.82 mM for tumor cells, and 0.75~0.79 mM for normal cells, yielding very low tumor-specificity (TS = 0.4–1.3), as compared with anticancer drugs (5-FU, melphalan, peplomycin) (TS = 4.1–9.7) [23]. Based on this finding, three human normal oral cells were exposed to 2 mM eugenol for up to 100 min. 

**Figure 9:**
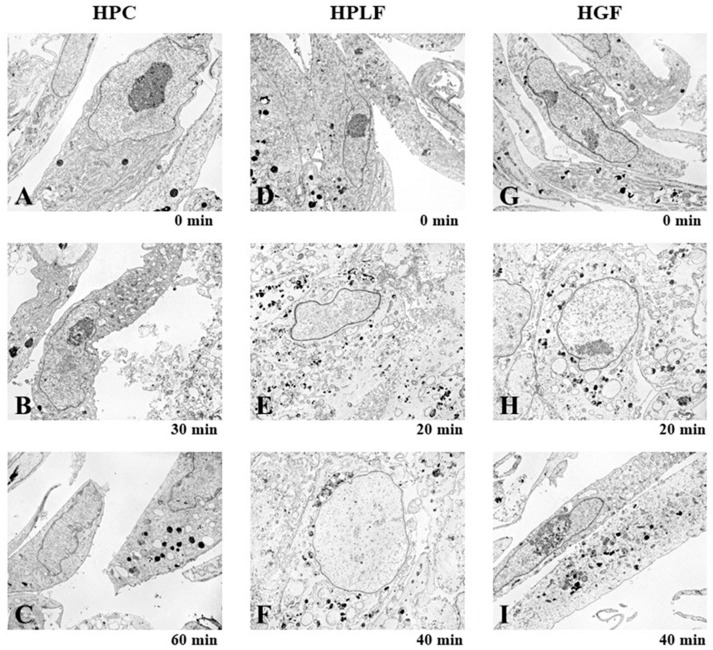
Rapid changes in the mitochondria and endoplasmic reticulum in normal oral cells by eugenol. Human normal oral HCP (**A**–**C**), HPLF (**D**–**F**), and HGF (**G**–**I**) cells were exposed to eugenol (2 mM) for the indicated times, and then subjected to TEM analysis. Images magnified at ×3000.

Exposure of human normal oral cells (HPC, HPLF, HPC) to eugenol for only 20~60 min produced changes in mitochondria and endoplasmic reticulum, inducing mitochondrial collapse, vacuolization, and secondary lysosome (Figure 9). 

#### 3.2.2. Eugenol Rapidly Suppressed TCA Cycle

**Figure 10:**
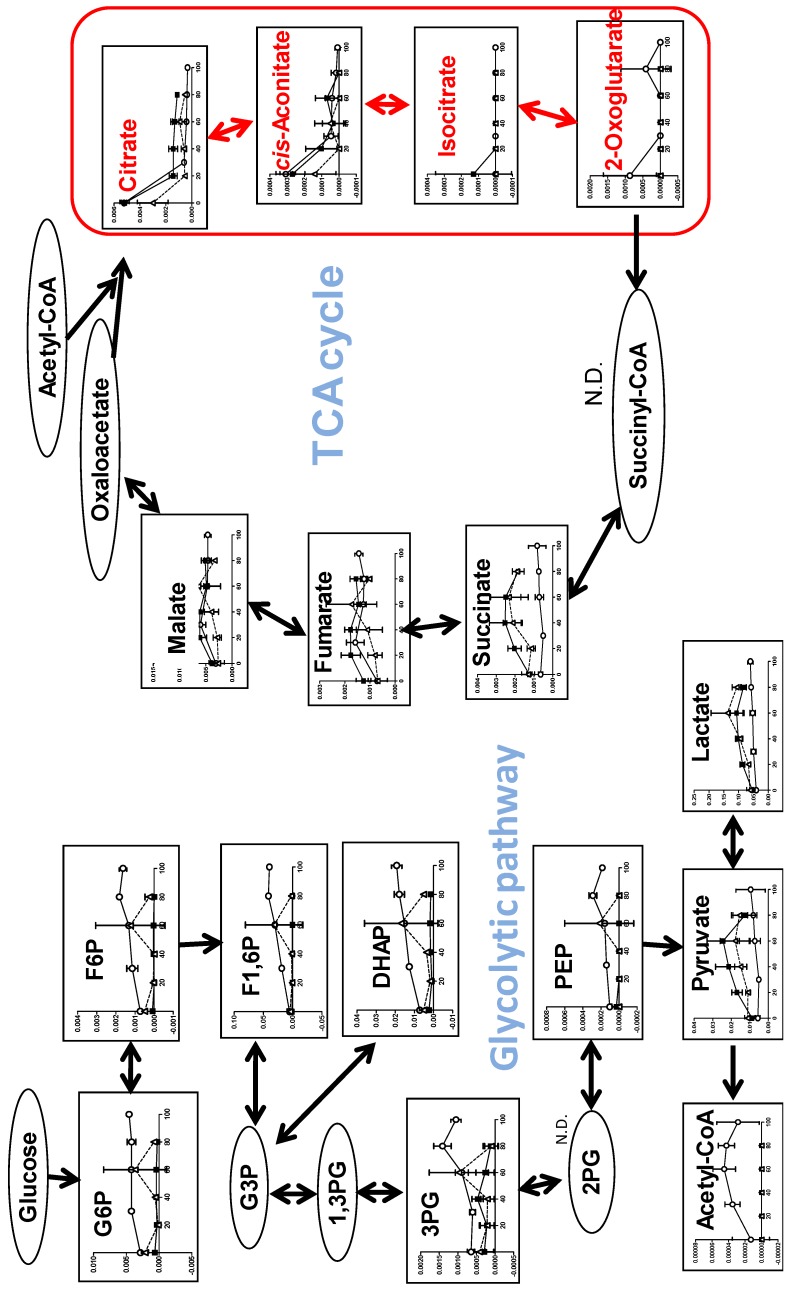
Eugenol suppressed the TCA cycle. HPC (〇), HPLF (■), HGF (△). Vertical axis, μmol/cell. Three groups were compared by one-way ANOVA (no post test).

The most dramatic changes to the TCA cycle were observed after treatment with eugenol (Figure 10). Within 20 min exposure to eugenol, intracellular concentrations of citrate, *cis*-aconitate, isocitrate, and 2-oxoglutarate rapidly declined in all three cells, whereas that of succinate, fumarate, and malate maintained almost constant levels over 80~100 min, indicating the rapid suppression of TCA cycle progression. On the other hand, eugenol treatment did not reduce, but rather slightly increased, the intracellular concentration of glycolytic metabolites (G6P, F6P, F1,6P, DHAP, 3PG, PEP, acetyl CoA, pyruvate, lactate) (Figure 10).

**Figure 11:**
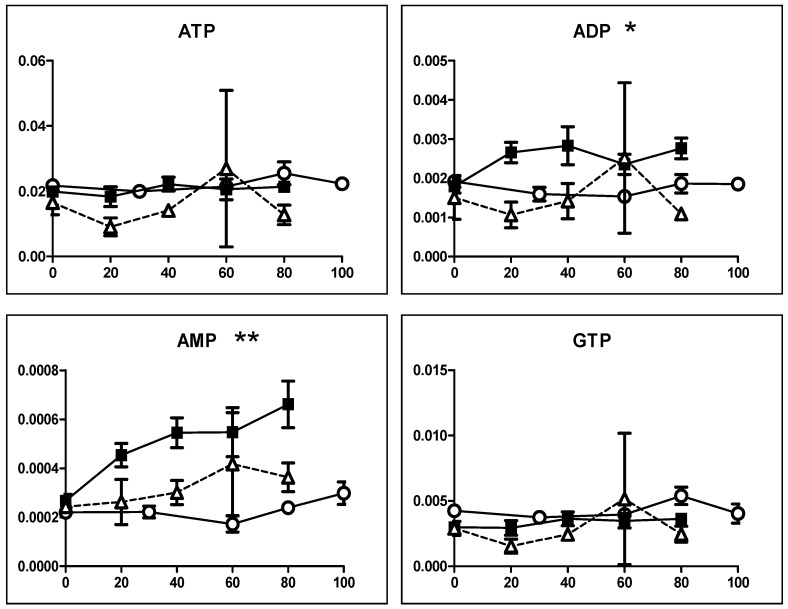
Eugenol generally does not affect the ATP concentrations. HPC (〇), HPLF (■), HGF (△). Vertical axis, μmol/cell. Three groups were compared by one-way ANOVA (no post test). * *p* < 0.05, ** *p* < 0.01.

Eugenol treatment did not apparently affect the intracellular concentration of ATP in all three cells (Figure 11). Conversion of ATP to ADP was approximately 10%, and that of ADP to AMP was approximately 10% in all cells, indicating the very low incidence of ATP utilization (AMP/ATP = 0.01).

Similarly, eugenol treatment slightly increased the intracellular concentration of 19 amino acids, except for cysteine, which was undetectable in all cells (Table S2).

## 4. Discussion

### 4.1. Inhibition of TCA Cycle by Benzaldehyde (BA) in Malignant Cells

The present study demonstrated that among three SBA-related compounds (SBA, BA, SA), BA showed the following three distinct properties from others. First, in terms of catalase-sensitivity, the cytotoxicity of BA against human OSCC cell line (HSC-2) was not affected by the addition of catalase, whereas that of SBA and SA was considerably reduced by catalase. This suggests that the release of hydrogen peroxide into the extracellular milieu is important for the cell death induction by SBA and SA, but not so in the cell death induced by BA. Since catalase does not penetrate inside the cells, the possibility that intracellular hydrogen oxide may play a role in the BA-induced cytotoxicity cannot be excluded at present. Second, BA showed an inability to induce vacuolization. Third, BA was shown to inhibit the TCA cycle, possibly due to the poor supply of pyruvate or β-alanine (and their precursors). The reduction of ATP utilization (as assessed by the ratio of AMP/ATP) further supports the non-apoptosis induction by BA [9]. Since BA showed the highest tumor-specificity among three SBA-related compounds, the inhibition of TCA cycle may be the target of anti-cancer therapy.

A closer look at the dose-response of SA revealed its biphasic effect. Lower concentrations (0.625, 1.25, 2.5 mM) of SA increased the intracellular ATP level, while higher concentrations (5, 10 mM) of SA reduced the ATP level. On the other hand, lower concentrations (0.625, 1.25, 2.5 mM) of SA reduced the intracellular concentration of AMP, adenosine, IMP, and inosine, while higher concentrations (5, 10 mM) of SA elevated their concentrations (Figure 7). This further supports the bi-phasic action of SA [12]. We have previously reported that millimolar concentrations of SA (which is a popular reducing agent) produced hydrogen peroxide, reduced intracellular GSH levels, and induced apoptosis in HL-60 human promyelocytic leukemia, in a Ca^2+^-dependent manner [13].

### 4.2. Inhibition of TCA Cycle by Eugenol in Non-Malignant Cells

We also found that eugenol (2 mM) induced the rapid suppression of the TCA cycle in all three human normal oral cells (HGF, HPLF, HPC). This was in nice contrast to our previous finding that eugenol slightly elevated the intracellular concentration of isocitrate and did not significantly change that of 2-oxoglutarate [33]. This demonstrated that eugenol reduced the TCA cycle only in normal oral cells, but not in OSCC cells. The present study further confirmed that eugenol induced non-apoptotic cell death, since eugenol did not affect ATP utilization except in HPLF cells (Figure 10). At present, what type of cell death eugenol has induced is yet to be determined. Considering the induction of vacuolization by eugenol, paraptosis (which causes cytoplasmic vacuolization and mitochondria enlargement [41]) may have been induced. Alternatively, considering the pro-inflammatory action of eugenol reported against HGF [42], pyroptosis (called cell inflammatory necrosis), characterized by swelling of the cell, the release of cell contents, and pro-inflammatory cytokines [43] may also be involved. Since natural products can modulate many types of cell death against cancer, such as paraptosis, necroptosis, mitotic catastrophe, and so on [44], further studies are necessary to identify the type of cell death that eugenol induces in normal oral cells. The narrow therapeutic range of eugenol suggests the importance of careful monitoring of its cytotoxicity against oral normal cells during dental treatment.

### 4.3. Combination Experiments with Anticancer Drugs

**Figure 12:**
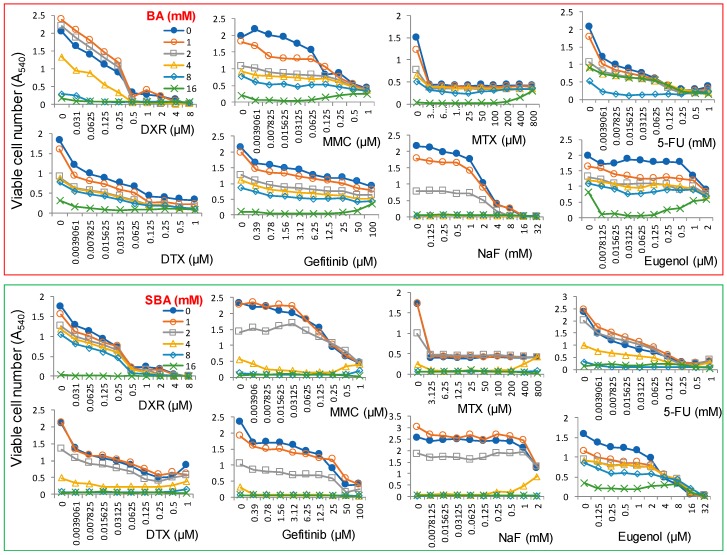
Combination effects of anticancer drugs and dental medicines on the augmentation of the cytotoxicity of BA (upper column) and SBA (lower column). HSC-2 cells were incubated for 48 h with the indicated concentrations of drugs in the presence of 0, 1, 2, 4, 8, or 16 mM BA or SBA, and the viable cell number was determined by MTT methods. Each value represents mean ± S.D. of triplicate assays. DXR, doxorubicin; MMC, mitomycin C; MTX, methotrexate; 5-FU, 5-fluorouracil; DTX, docetaxel; NaF, sodium fluoride.

We have started the search for anticancer drugs that enhance the cytotoxicity of BA against human OSCC cell line HSC-2 cells. We have previously reported the mean value of CC_50_ of several anticancer drugs against four human OSCC cell lines (Ca9-22, HSC-2, HSC-3, and HSC-4) and their tumor-specificity (TS), which was determined by the ratio of the mean value of CC_50_ against three normal human oral cells (HGF, HPC, and HPC) divided by the mean value of CC_50_ against four OSCC cell lines. The anticancer drugs that we tested for their CC_50_ and TS values are doxorubicin (CC_50_ = 0.09 µM, TS = 70), mitomycin C (CC_50_ = 1.3 µM, TS = 31), methotrexate (CC_50_ < 2.4 µM, TS > 170), 5-FU (CC_50_ = 99 µM, TS > 10), docetaxel (CC_50_ < 0.032 µM, TS > 2708), and gefitinib (CC_50_ = 17 µM, TS = 4) [45]. We found that simultaneous addition of doxorubicin, mitomycin C, methotrexate, 5-FU, docetaxel and gefitinib, and dental medicines such as sodium fluoride and eugenol did not show synergistic augmentation of the cytotoxicity of BA and SBA against HSC-2 cells (Figure 12). Further study is needed to find the optimal condition for enhancing the cytotoxicity of BA against OSCC cells.

In conclusion, both BA and eugenol suppressed the TCA cycle at the early stage of non-apoptotic cell death. As compared with eugenol, BA did not induce vacuolization, inhibited growth more slowly, and had higher tumor-specificity. Further studies are needed to design a methodology that enhances the antitumor potential of BA and reduces the cytotoxicity of eugenol in order to allow for safe clinical application. Also, the in vivo metabolic fate of SBA should be monitored for its clinical use.

## Figures and Tables

**Table 1:** Biological activities of SBA and its cleaved products, BA and SA.

Biological Activities	SBA	SA	BA	Ref.
Antitumor activity (in vivo)	Yes	No	Yes	[5]
Tumor-specificity (TS = CC_50_ (normal)/CC_50_ (tumor))	2	2.5	8.8	[8,9]
Apoptosis-induction in HSC-2 cells	No	N.D.	No	[8,9]
Apoptosis-induction in HL-60 cells	Yes	Yes	No	[10]
Cytotoxicity by addition of copper	Increase	Increase	N.D.	[11,12]
Cytotoxicity by addition of iron, cysteine analog, catalase	Not clear	Decrease	N.D.	[11,12,13]
Radical generation	Yes	Yes	N.D.	[11,12]
Oxidation potential, H_2_O_2_ production, methionine oxidation	Yes	Yes	N.D.	[11,12,13,14,15]

N.D., not determined.

## Supplementary Materials

The following are available online at http://www.mdpi.com/2305-6320/5/4/116/s1, Figure S1: Effect of SBA, BA and SA on Pyrimidine metabolism (A), puring metabolism (B), glycolysis (C), TCA cycle (D), pentose phosphate pathway (E), arginine, proline metabolism and urea cycle (F), glycine, serine and threonine metabolism (G), alanine, asparagine and aspartic acid metabolism, β-alanine biosynthesis and metabolism (H), lysine metabolisum (I), methionine and cysteine metabolism (G), histidine metabolism (K), tryptophane metabolism (L), phenylalanine, tyrosine metabolism (M), valine, leucine and isoleucine metabolism (N) and GABA biosynthesis and metabolism, glutamine metabolism (O) in HSC-2 cells. Table S1: Metabolic changes induced by SBA, BA and SA (A). 171 compounds were detected. Each value represents amol/cell, and can be seen by magnifier. Table S2: Metabolic changes induced by SBA, BA in HSC-2 cells (Exp. I), and by eugenol in HGF, HPC and HPLF cells (Exp. II). 188 compounds were detected. Each value represents amol/cell, and can be seen by magnifier.

## References

[1] Takeuchi S., Kochi M., Sakaguchi K., Nakagawa K., Mizutani T. (1978). Benzaldehyde as a carcinostatic principle in figs. Agric. Biol. Chem..

[2] Kochi M., Takeuchi S., Mizutani T., Mochizuki K., Matsumoto Y., Saito Y. (1980). Antitumor activity of benzaldehyde. Cancer Treat. Rep..

[3] Kochi M., Isono N., Niwayama M., Shirakabe K. (1985). Antitumor activity of a benzaldehyde derivative. Cancer Treat. Rep..

[4] Kochi M., Ueda S., Hagiwara T., Bresciani F., King R.J.B. (1988). Antitumor activity of sodium benzylidenascorbate. Progress in Cancer Research and Therapy 35: Hormones and Cancer 3.

[5] Sakagami H., Asano K., Fukuchi K., Gomi K., Ota H., Kazama K., Tanuma S., Kochi M. (1991). Induction of tumor degeneration by sodium benzylideneascorbate. Anticancer Res..

[6] Sakagami H., Sakagami T., Takeda M., Iwaki K., Takeda K. (1993). Determination of sodium 5,6-benzylidene-l-ascorbate and related compounds by high-performance liquid chromatography. J. Chromatogr. Coruña.

[7] Sakagami H., Sakagami T., Yamamura M., Takahashi H., Shibuya I., Takeda M. (1995). Stability of sodium 5,6-benzylidene-l-ascorbate. Anticancer Res..

[8] Kishino K., Hashimoto K., Amano O., Kochi M., Sakagami H. (2008). Tumor-specific cytotoxicity and type of cell death induced by sodium 5,6-benzylidene-l-ascorbate. Anticancer Res..

[9] Ariyoshi-Kishino K., Hashimoto K., Amano O., Kochi M., Sakagami H. (2010). Tumor-specific cytotoxicity and type of cell death induced by benzaldehyde. Anticancer Res..

[10] Kuribayashi N., Sakagami H., Sakagami T., Niimi E., Shiokawa D., Ikekita M., Takeda M., Tanuma S. (1994). Induction of DNA fragmentation in human myelogenous leukemic cell lines by sodium 5,6-benzylidene-l-ascorbate and its related compounds. Anticancer Res..

[11] Sakagami H., Satoh K., Kochi M. (1997). Comparative study of the antitumor action between sodium 5,6-bnzylidene-l-ascorbate and sodium ascorbate (Minireview). Anticancer Res..

[12] Sakagami H., Satoh K., Hakeda Y., Kumegawa M. (2000). Apoptosis-inducing activity of vitamin C and vitamin K. Cell Mol. Biol..

[13] Sakagami H., Kuribayashi N., Iida M., Hagiwara T., Takahashi H., Yoshida H., Shiota F., Ohata H., Momose K., Takeda M. (1996). The requirement for and mobilization of calcium during induction by sodium ascorbate and by hydrogen peroxide of cell death. Life Sci..

[14] Sakagami H., Satoh K., Kadofuku T., Takeda M. (1997). Methionine oxidation and apoptosis induction by ascorbate, gallate and hydrogen peroxide. Anticancer Res..

[15] Sakagami H., Hosaka M., Arakawa H., Maeda M., Satoh K., Ida Y., Asano K., Hisamitsu T., Takimoto M., Ota H. (1998). Role of hydrogen peroxide in antitumor activity induction by sodium 5,6-benzylidene-L-ascorbate. Anticancer Res..

[16] Sweet C. (1930). Procedure for treatment of exposed and pulpless deciduous teeth. J. Am. Dent. Assoc..

[17] Primosch R.E. (1997). Primary tooth pulp therapy as taught in predoctoral pediatric dental programs in the United States. Pediatr. Dent..

[18] Hume W. (1984). An analysis of the release and the diffusion through dentin of eugenol from zinc oxide-eugenol mixtures. J. Dent. Res..

[19] Glass R., Zander H. (1949). Pulp healing. J. Dent. Res..

[20] Barja-Fidalgo F., Moutinho-Ribeiro M., Oliveira M.A.A., de Oliveira B.H. (2011). A systematic review of root canal filling materials for deciduous teeth: Is there an alternative for zinc oxide-eugenol?. ISRN Dent..

[21] Hilton T.J. (2009). Keys to clinical success with pulp capping: A review of the literature. Oper. Dent..

[22] Hume W.R. (1984). Effect of eugenol on respiration and division in human pulp, mouse fibroblasts, and liver cells in vitro. J. Dent. Res..

[23] Koh T., Machino M., Murakami Y., Umemura N., Sakagami H. (2013). Cytotoxicity of dental compounds against human oral squamous cell carcinoma and normal oral cells. In Vivo.

[24] Atsumi T., Fujisawa S., Satoh K., Sakagami H., Iwakura I., Ueha T., Sugita Y., Yokoe I. (2000). Cytotoxicity and radical intensity of eugenol, isoeugenol or related dimers. Anticancer Res..

[25] Jaqanathan S.K., Mazumdar A., Mondhe D., Mandal M. (2011). Apoptotic effect of eugenol in human colon cancer cell lines. Cell Biol. Int..

[26] Vidhya N., Devaraj S.N. (2011). Induction of apoptosis by eugenol in human breast cancer cells. Indian J. Exp. Biol..

[27] Majno G., Joris I. (1995). Apoptosis, oncosis, and necrosis. An overview of cell death. Am. J. Pathol..

[28] Halestrap A.P., Clarke S.J., Javadov S.A. (2004). Mitochondrial permeability transition pore opening during myocardial reperfusion—A target for cardioprotection. Cardiovasc. Res..

[29] Halestrap A. (2005). Biochemistry: A pore way to die. Nature.

[30] Otsuki S., Morshed S.R.M., Chowdhury S.A., Takayama F., Satoh T., Hashimoto K., Sugiyama K., Amano O., Yasui T., Yokote Y. (2005). Possible link between glycolysis and apoptosis induced by sodium fluoride. J. Dent. Res..

[31] Otsuki S., Sugiyama K., Amano O., Yasui T., Sakagami H. (2011). Negative regulation of NaF-induced apoptosis by Bad-CAII complex. Toxicology.

[32] Sakagami H., Sugimoto M., Tanaka S., Onuma H., Ota S., Kaneko M., Soga T., Tomita M. (2014). Metabolomic profiling of sodium fluoride-induced cytotoxicity in an oral squamous cell carcinoma cell line. Metabolomics.

[33] Koh T., Murakami Y., Tanaka S., Machino M., Onuma H., Kaneko M., Sugimoto M., Soga T., Tomita M., Sakagami H. (2013). Changes of metabolic profiles in an oral squamous cell carcinoma cell line induced by eugenol. In Vivo.

[34] Kantoh K., Ono M., Nakamura Y., Nakamura Y., Hashimoto K., Sakagami H., Wakabayashi H. (2010). Hormetic and anti-radiation effects of tropolone-related compounds. In Vivo.

[35] Sakagami H., Shimada C., Kanda Y., Amano O., Sugimoto M., Ota S., Soga T., Tomita M., Sato A., Tanuma S. (2015). Effects of 3-styrylchromones on metabolic profiles and cell death in oral squamous cell carcinoma cells. Toxocol. Rep..

[36] Garcia-Contreras1 R., Sugimoto M., Umemura N., Kaneko M., Hatakeyama Y., Soga T., Tomita M., Scougall-Vilchis R.J., Contreras-Bulnes R., Nakajima H. (2015). Alteration of metabolomic profiles by titanium dioxide nanoparticles in human gingivitis model. Biomaterials.

[37] Soga T., Baran R., Suematsu M., Ueno Y., Ikeda S., Sakurakawa T., Kakazu Y., Ishikawa T., Robert T., Nishioka T. (2006). Differential metabolomics reveals ophthalmic acid as an oxidative stress biomarker indicating hepatic glutathione consumption. J. Biol. Chem..

[38] Sugimoto M., Sakagami H., Yokote Y., Onuma H., Kaneko M., Mori M., Sakaguchi Y., Soga T., Tomita M. (2012). Non-targeted metabolite profiling in activated macrophage secretion. Metabolomics.

[39] Sugimoto M., Wong D.T., Hirayama A., Soga T., Tomita M. (2010). Capillary electrophoresis mass spectrometry-based saliva metabolomics identified oral, breast and pancreatic cancer-specific profiles. Metabolomics.

[40] Sugimoto M., Kawakami M., Robert M., Soga T., Tomita M. (2012). Bioinformatics tools for mass spectroscopy-based metabolomic data processing and analysis. Curr. Bioinform..

[41] Sugimori N., Espinoza J.L., Trung L.Q., Takami A., Kondo Y., An D.T., Sasaki M., Wakayama T., Nakao S. (2015). Paraptosis cell death induction by the thiamine analog benfotiamine in leukemia cells. PLoS ONE.

[42] Koh T., Murakami Y., Tanaka S., Machino M., Sakagami H. (2013). Re-evaluation of anti-inflammatory potential of eugenol in IL-1β-stimulated gigngival fibroblast and pulp cells. In Vivo.

[43] Ma Y., Jiang J., Gao Y., Shi T., Zhu X., Zhang K., Lu K., Xue B. (2018). Research progress of the relationship between pyroptosis and disease. Am. J. Transl. Res..

[44] Guamán-Ortiz L.M., Orellana M.I., Ratovitski E.A. (2017). Natural compounds as modulators of non-apoptotic cell death in cancer cells. Curr. Genom..

[45] Sakagami H., Okudaira N., Masuda Y., Amano O., Yokose S., Kanda Y., Suguro M., Natori T., Oizumi H., Oizumi T. (2017). Induction of apoptosis in human oral keratinocyte by doxorubicin. Anticancer Res..

