# Supplementary material for: Changes in Metabolic Profiles of Human Oral Cells by Benzylidene Ascorbates and Eugenol
Source: Medicines (Basel). 2018 Oct 31;5(4):116. doi: 10.3390/medicines5040116 (PMC6313480; doi:10.3390/medicines5040116)
Supplement: Supplementary file 1 [file medicines-05-00116-s001.pdf]

Supplementary Materials: Changes in Metabolic Profiles of Human Oral Cells by Benzylidene Ascorbates and Eugenol

Hiroshi Sakagami, Masahiro Sugimoto, Yumiko Kanda, Yukio Murakami, Osamu Amano, Junko Saito and Atsuko Kochi

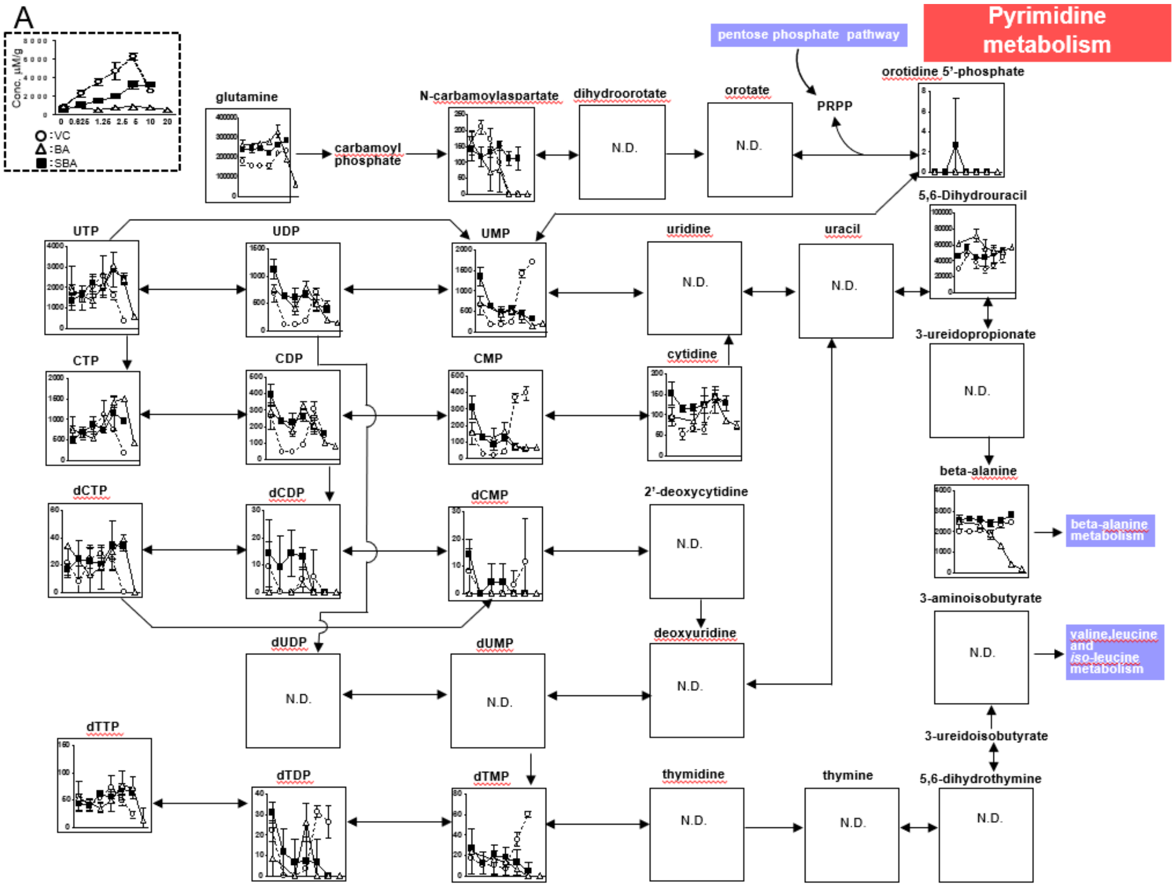

B

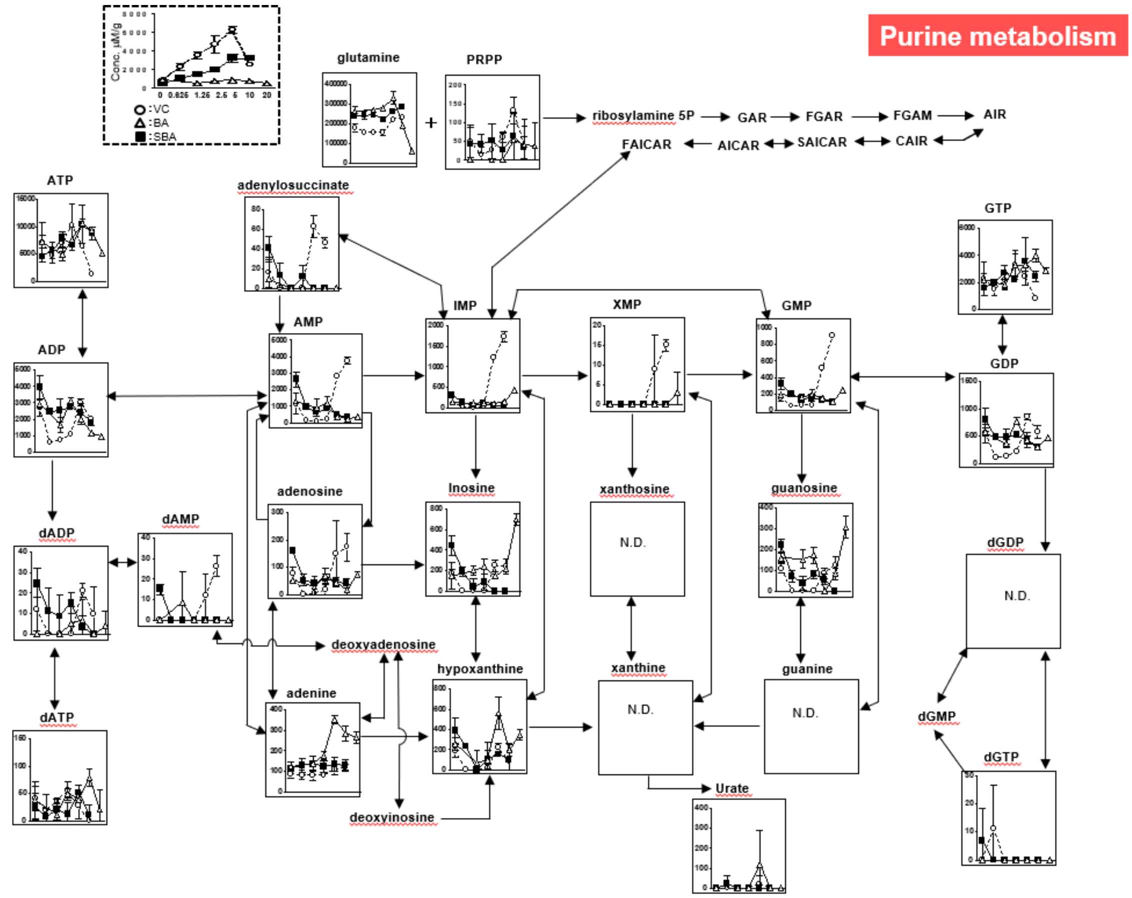
Purine metabolism
Conc. µM/g
:VC
:BA
:SBA
glutamine
PRPP
ribosylamine 5P
GAR
FGAR
FGAM
AIR
FAICAR
AICAR
SAICAR
CAIR
ATP
adenylosuccinate
GTP
IMP
XMP
GMP
ADP
AMP
GDP
adenosine
Inosine
xanthosine
guanosine
N.D.
dGDP
dADP
dAMP
deoxyadenosine
hypoxanthine
xanthine
guanine
adenine
dGMP
dATP
dGTP
deoxyinosine
Urate

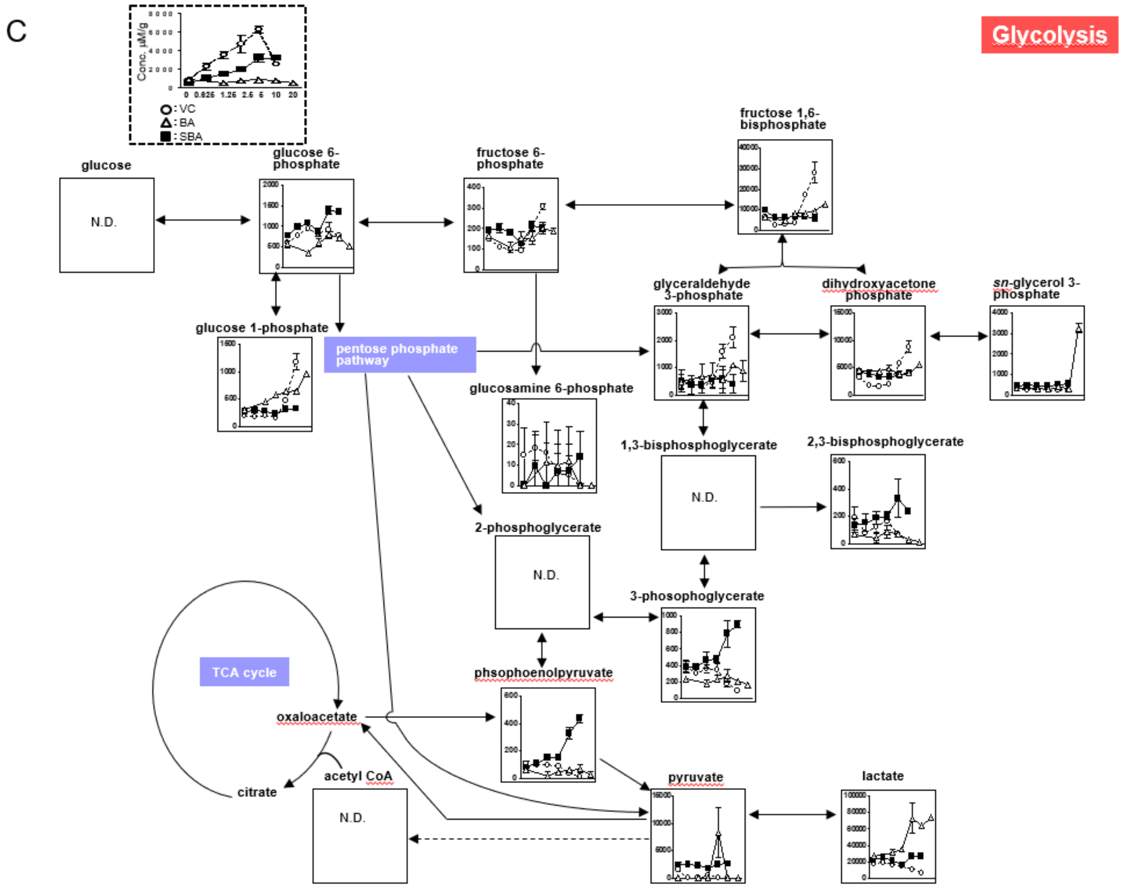
C
Glycolysis
Conc. µM/g
0 0.625 1.25 2.5 5 10 20
: VC
: BA
: SBA
glucose
N.D.
glucose 6-phosphate
fructose 6-phosphate
fructose 1,6-bisphosphate
glucose 1-phosphate
pentose phosphate pathway
glyceraldehyde 3-phosphate
dihydroxyacetone phosphate
sn-glycerol 3-phosphate
glucosamine 6-phosphate
1,3-bisphosphoglycerate
2,3-bisphosphoglycerate
2-phosphoglycerate
3-phosophoglycerate
TCA cycle
phsophoenolpyruvate
oxaloacetate
acetyl CoA
citrate
pyruvate
lactate

D

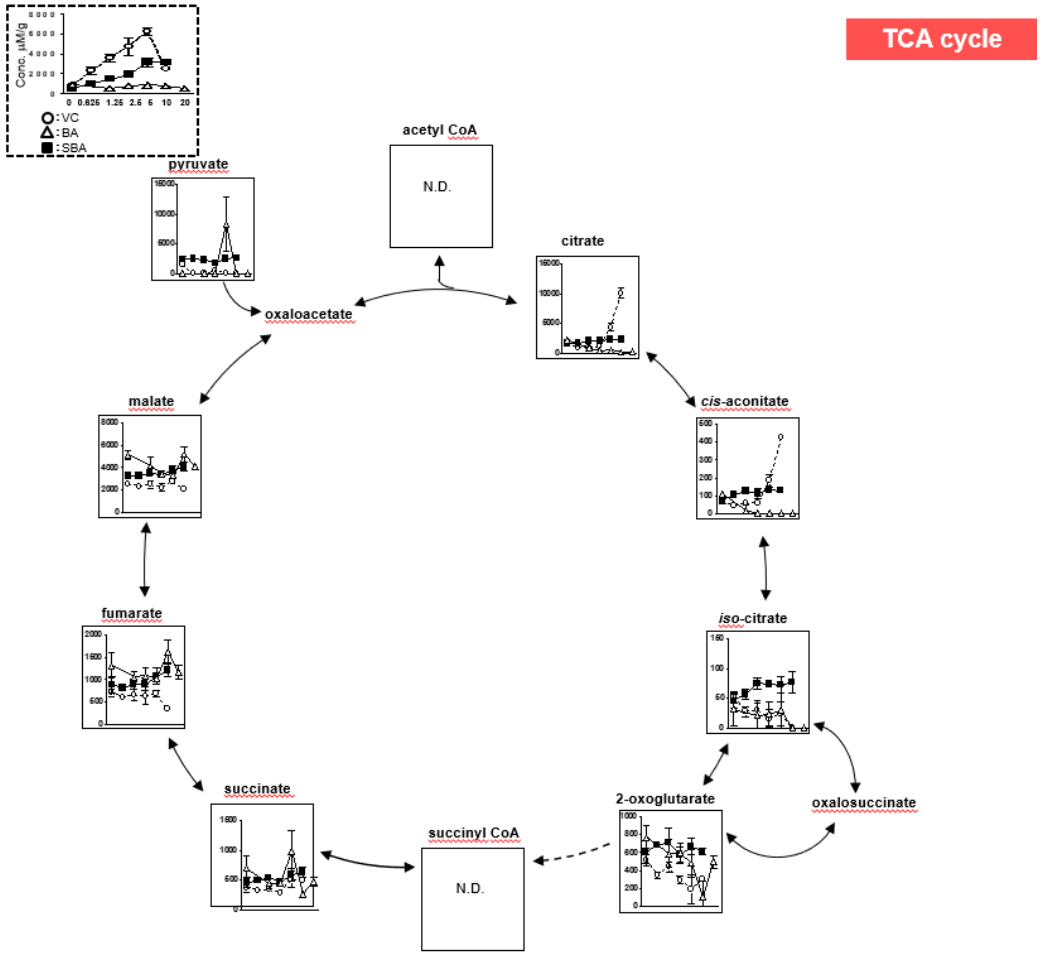
TCA cycle
Conc. μM/g
0 0.625 1.25 2.5 5 10 20
: VC
: BA
: SBA
pyruvate
acetyl CoA
N.D.
citrate
oxaloacetate
malate
cis-aconitate
fumarate
iso-citrate
succinate
succinyl CoA
N.D.
2-oxoglutarate
oxalosuccinate

E

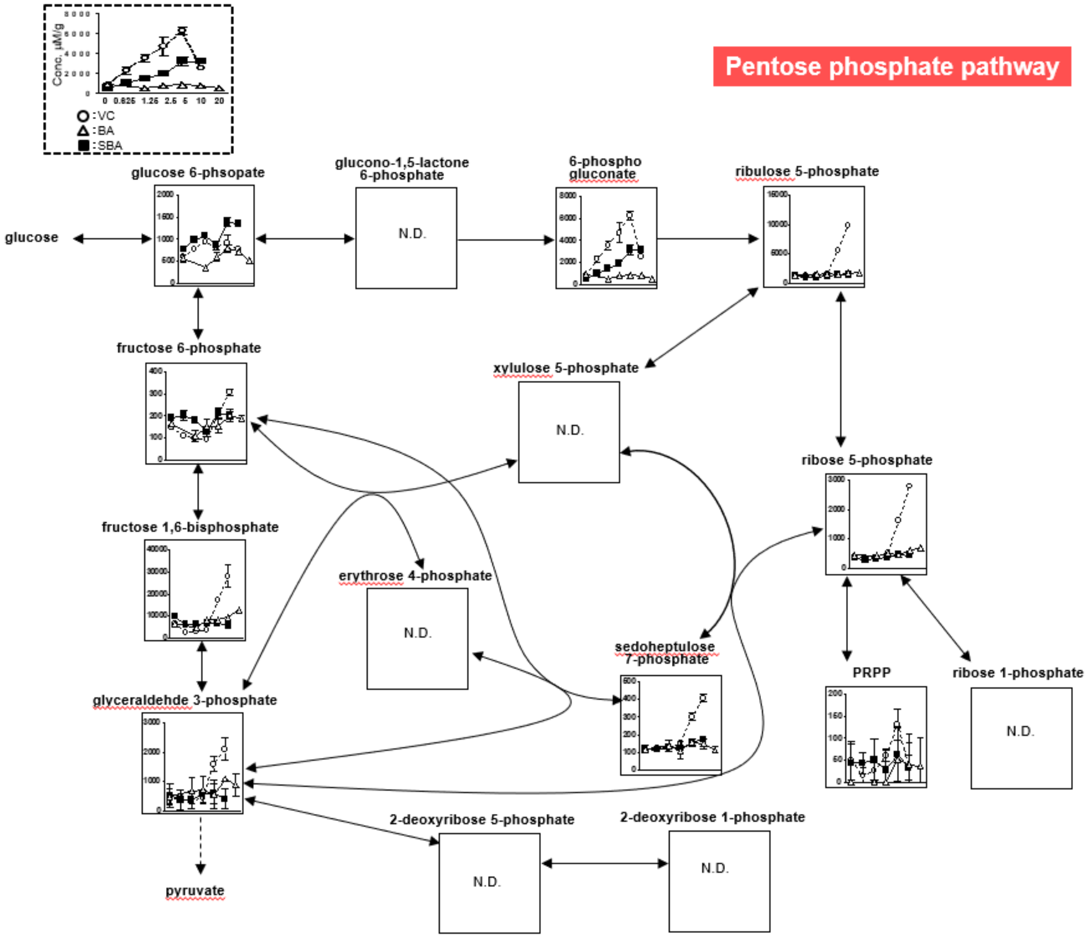
Pentose phosphate pathway
Conc. μM/g
0 0.625 1.25 2.5 5 10 20
: VC
: BA
: SBA
glucose
glucose 6-phsopate
glucono-1,5-lactone 6-phosphate
N.D.
6-phospho gluconate
ribulose 5-phosphate
fructose 6-phosphate
xylulose 5-phosphate
N.D.
ribose 5-phosphate
fructose 1,6-bisphosphate
erythrose 4-phosphate
N.D.
sedoheptulose 7-phosphate
PRPP
ribose 1-phosphate
N.D.
glyceraldehde 3-phosphate
2-deoxyribose 5-phosphate
N.D.
2-deoxyribose 1-phosphate
N.D.
pyruvate

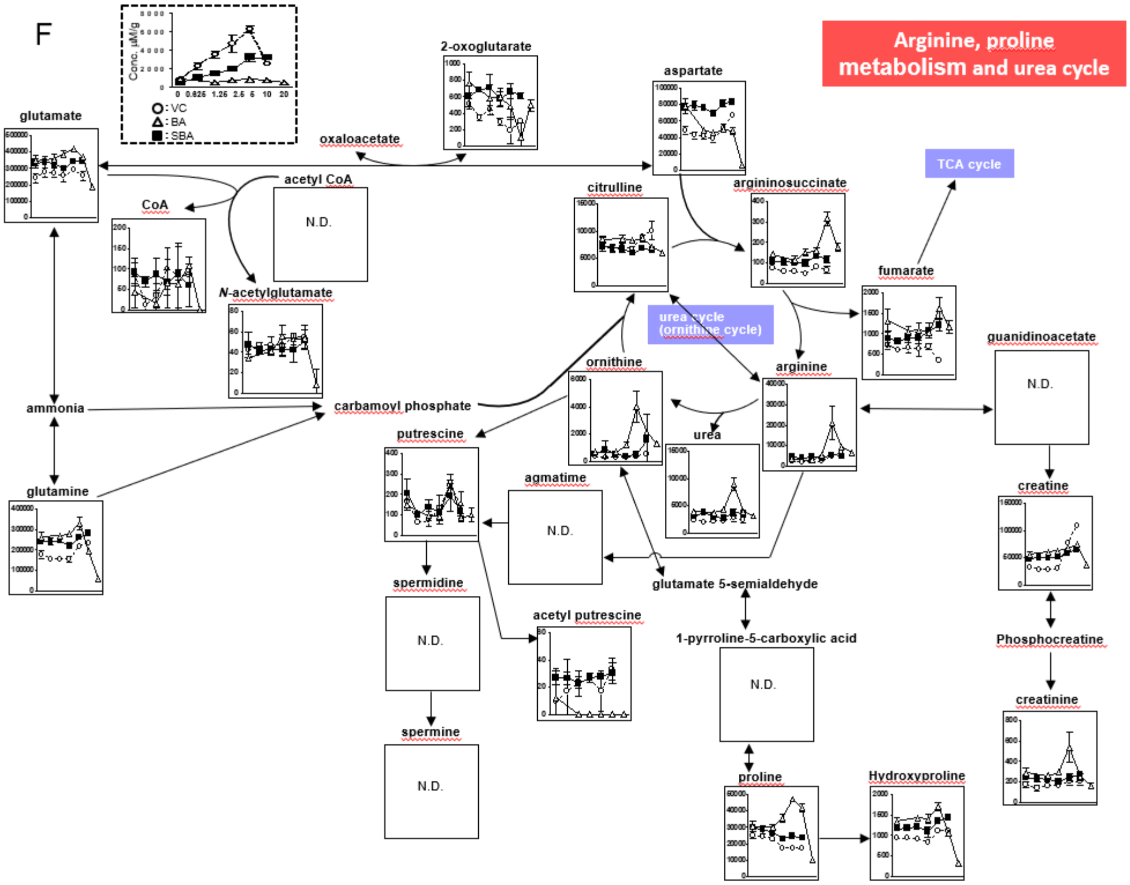
F
Conc. µM/g
0 0.625 1.25 2.5 5 10 20
: VC
: BA
: SBA
Arginine, proline metabolism and urea cycle
TCA cycle
glutamate
2-oxoglutarate
aspartate
oxaloacetate
acetyl CoA
N.D.
CoA
citrulline
argininosuccinate
N-acetylglutamate
fumarate
urea cycle (ornithine cycle)
guanidinoacetate
N.D.
ornithine
arginine
ammonia
carbamoyl phosphate
putrescine
urea
agmatime
N.D.
creatine
glutamine
glutamate 5-semialdehyde
spermidine
N.D.
acetyl putrescine
1-pyrroline-5-carboxylic acid
N.D.
Phosphocreatine
spermine
N.D.
creatinine
proline
Hydroxyproline

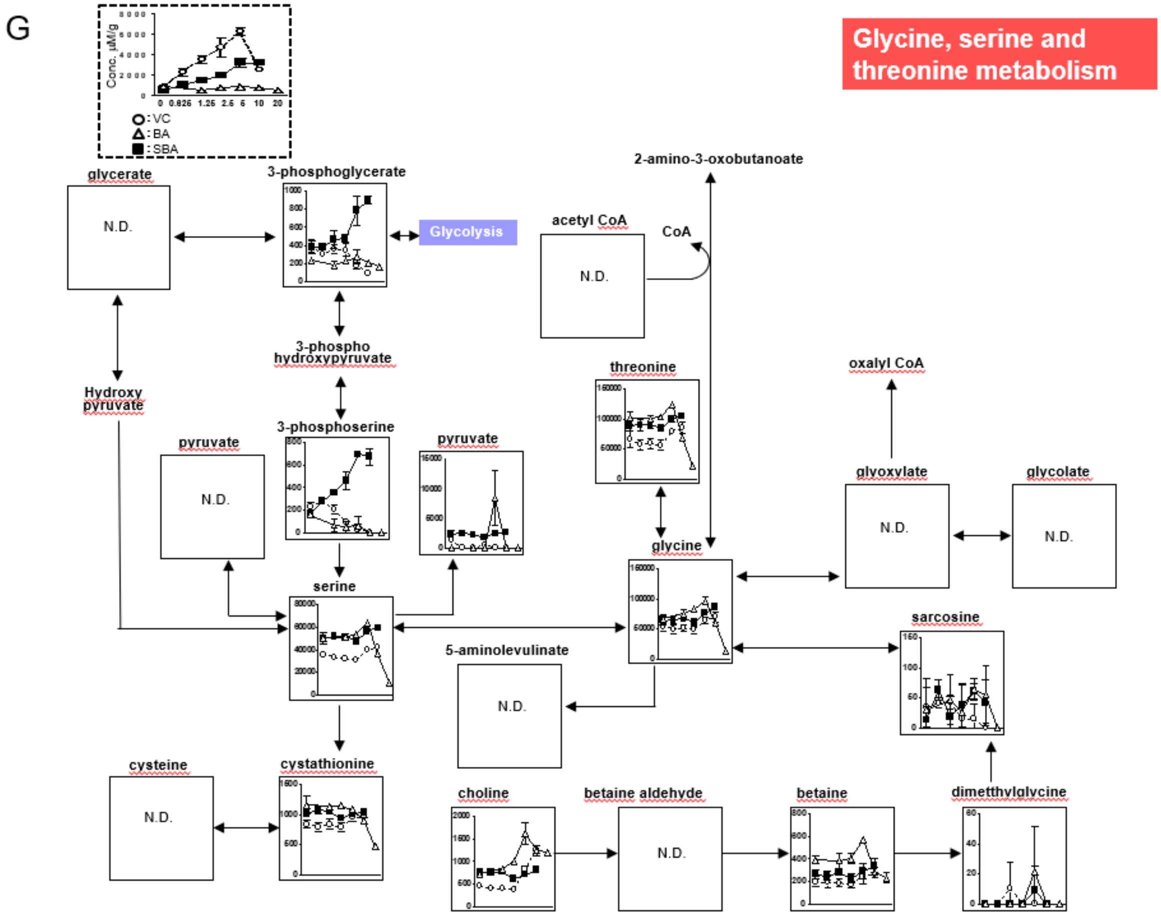
G
Conc. μM/g
O : VC
Δ : BA
■ : SBA
Glycine, serine and threonine metabolism
glycerate
N.D.
3-phosphoglycerate
Glycolysis
2-amino-3-oxobutanoate
acetyl CoA
N.D.
CoA
3-phospho hydroxypyruvate
Hydroxy pyruvate
3-phosphoserine
pyruvate
N.D.
pyruvate
threonine
oxalyl CoA
glyoxylate
N.D.
glycolate
N.D.
glycine
serine
5-aminolevulinate
N.D.
sarcosine
cysteine
N.D.
cystathionine
choline
betaine aldehyde
N.D.
betaine
dimethylglycine

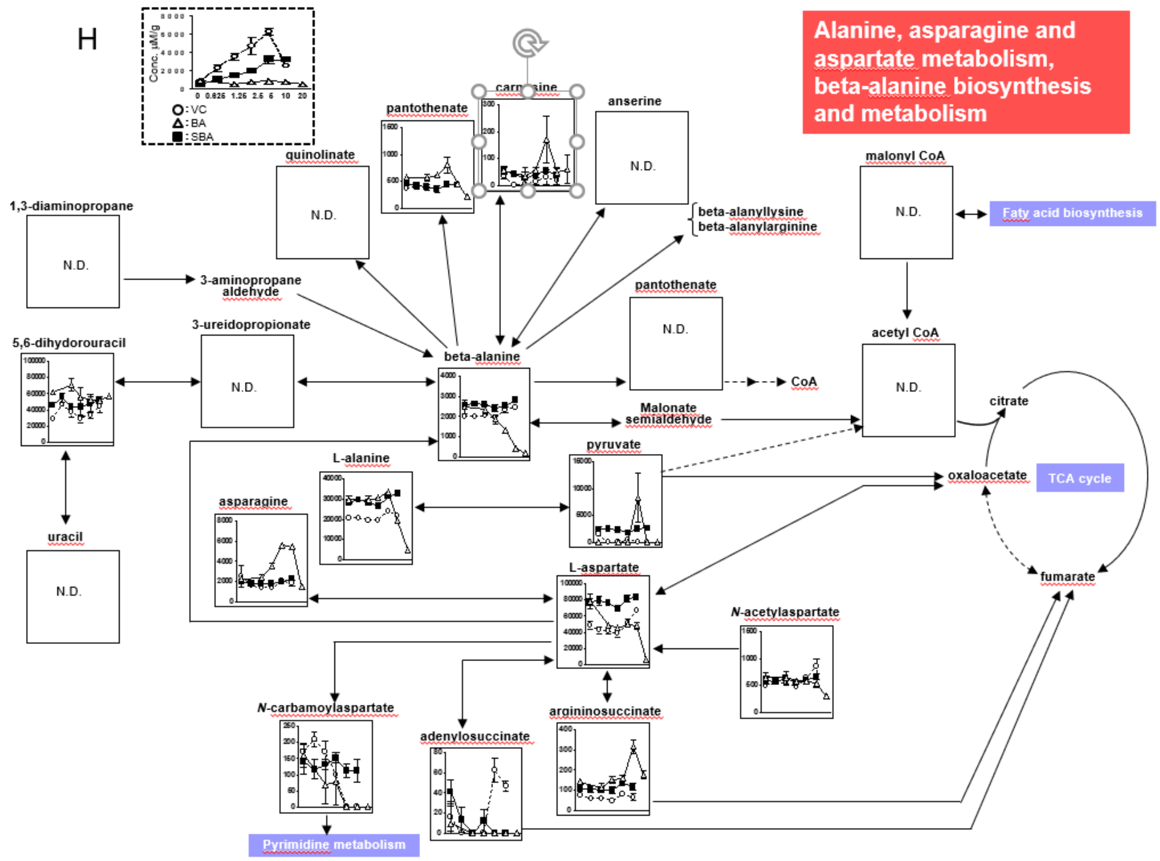
H
Alanine, asparagine and aspartate metabolism, beta-alanine biosynthesis and metabolism
Conc. μM/g
0 0.625 1.25 2.5 5 10 20
: VC
: BA
: SBA
quinolinate
pantothenate
carnosine
anserine
malonyl CoA
Faty acid biosynthesis
1,3-diaminopropane
3-aminopropane aldehyde
beta-alanyllysine
beta-alanylarginine
pantothenate
3-ureidopropionate
5,6-dihydorouracil
beta-alanine
CoA
acetyl CoA
Malonate semialdehyde
citrate
uracil
L-alanine
pyruvate
asparagine
oxaloacetate
TCA cycle
L-aspartate
fumarate
N-acetylaspartate
N-carbamoylaspartate
adenylosuccinate
argininosuccinate
Pyrimidine metabolism
N.D.

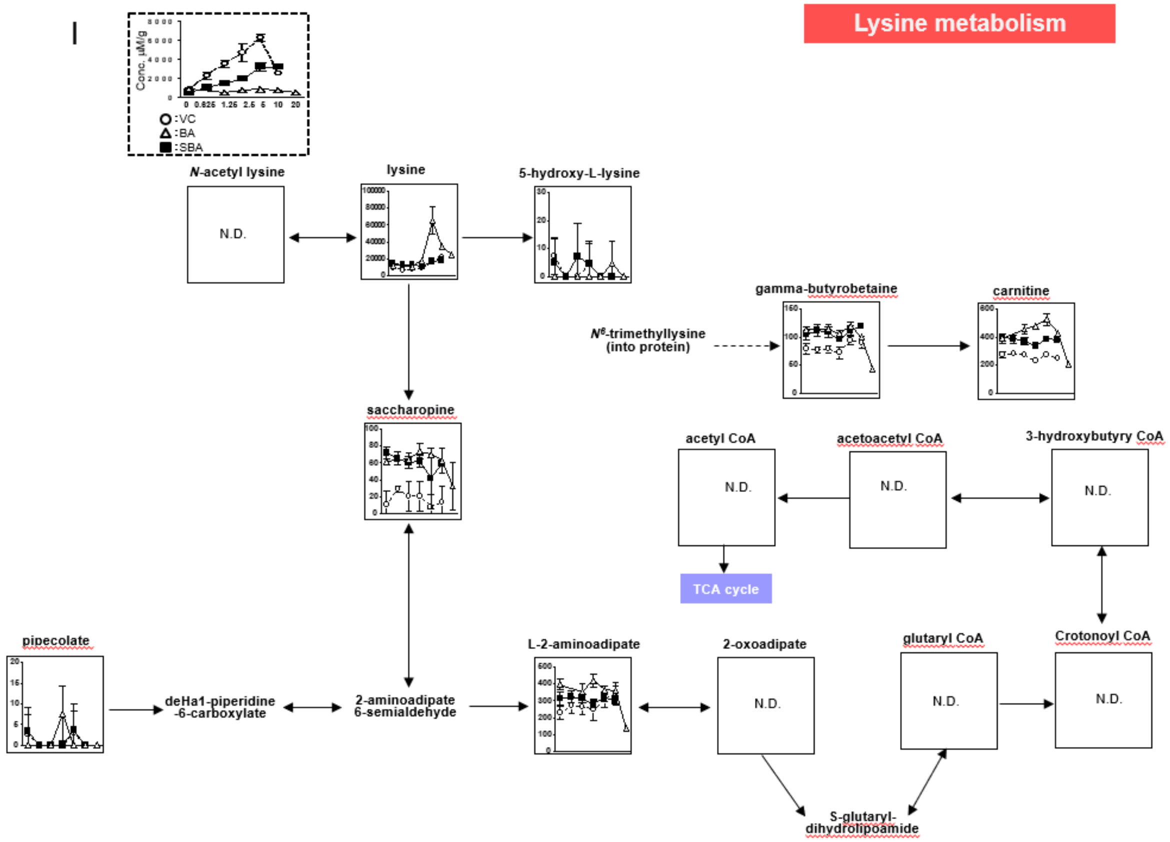
I
Lysine metabolism
Conc. μM/g
0 0.625 1.25 2.5 5 10 20
: VC
: BA
: SBA
N-acetyl lysine
lysine
5-hydroxy-L-lysine
gamma-butyrobetaine
carnitine
N6-trimethyllysine (into protein)
saccharopine
acetyl CoA
acetoacetyl CoA
3-hydroxybutyry CoA
TCA cycle
pipecolate
deHa1-piperidine -6-carboxylate
2-aminoadipate 6-semialdehyde
L-2-aminoadipate
2-oxoadipate
glutaryl CoA
Crotonoyl CoA
S-glutaryl-dihydrolipoamide
N.D.

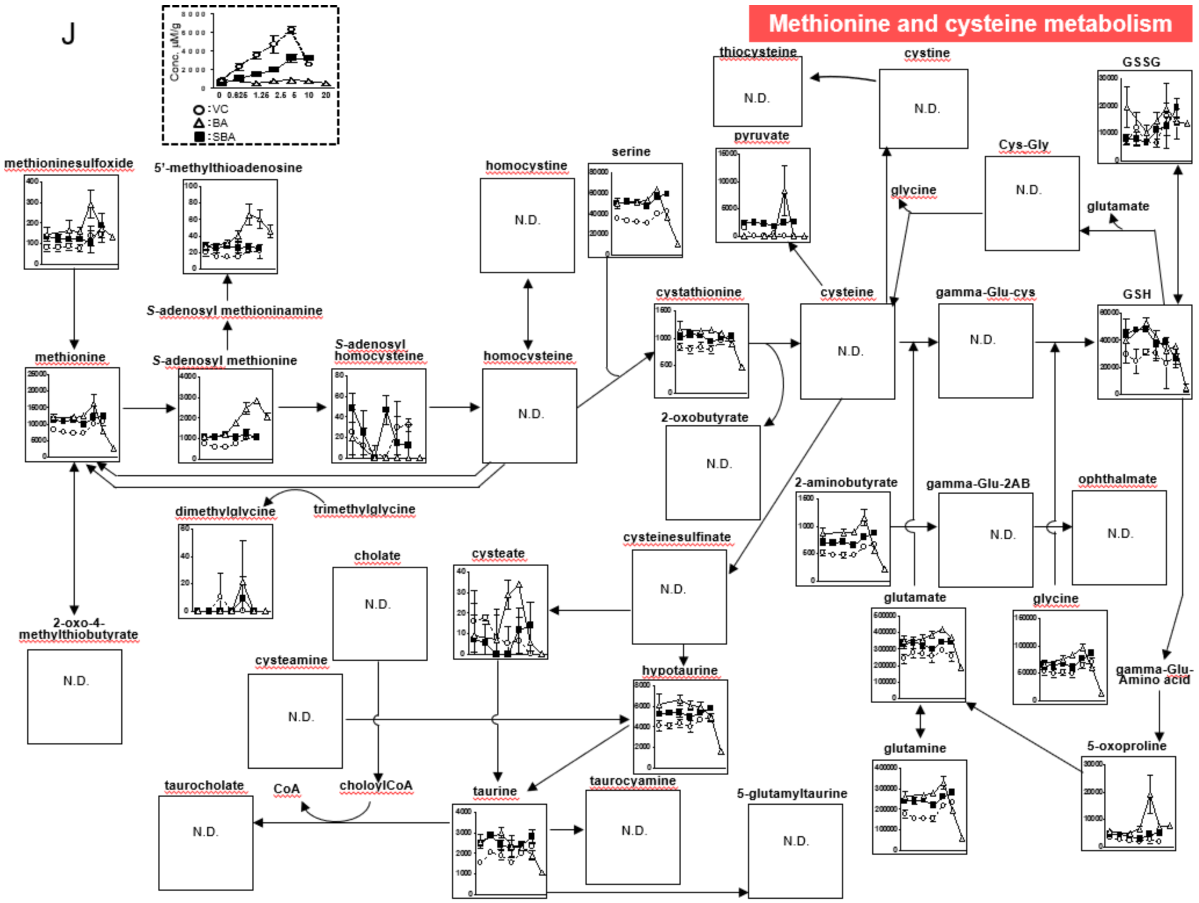
J
Conc. μM/g
:VC
:BA
:SBA
Methionine and cysteine metabolism
thiocysteine
N.D.
cystine
N.D.
GSSG
pyruvate
Cys-Gly
N.D.
glycine
glutamate
methioninesulfoxide
5'-methylthioadenosine
homocystine
N.D.
serine
S-adenosyl methioninamine
cystathionine
cysteine
N.D.
gamma-Glu-cys
N.D.
GSH
methionine
S-adenosyl methionine
S-adenosyl homocysteine
homocysteine
N.D.
2-oxobutyrate
N.D.
2-aminobutyrate
gamma-Glu-2AB
N.D.
ophthalmate
N.D.
dimethylglycine
trimethylglycine
cysteinesulfinate
N.D.
cholate
N.D.
cysteate
glutamate
glycine
2-oxo-4-methylthiobutyrate
N.D.
cysteamine
N.D.
hypotaurine
gamma-Glu-Amino acid
glutamine
5-oxoproline
taurocholate
N.D.
CoA
choloylCoA
taurine
taurocyamine
N.D.
5-glutamyltaurine
N.D.

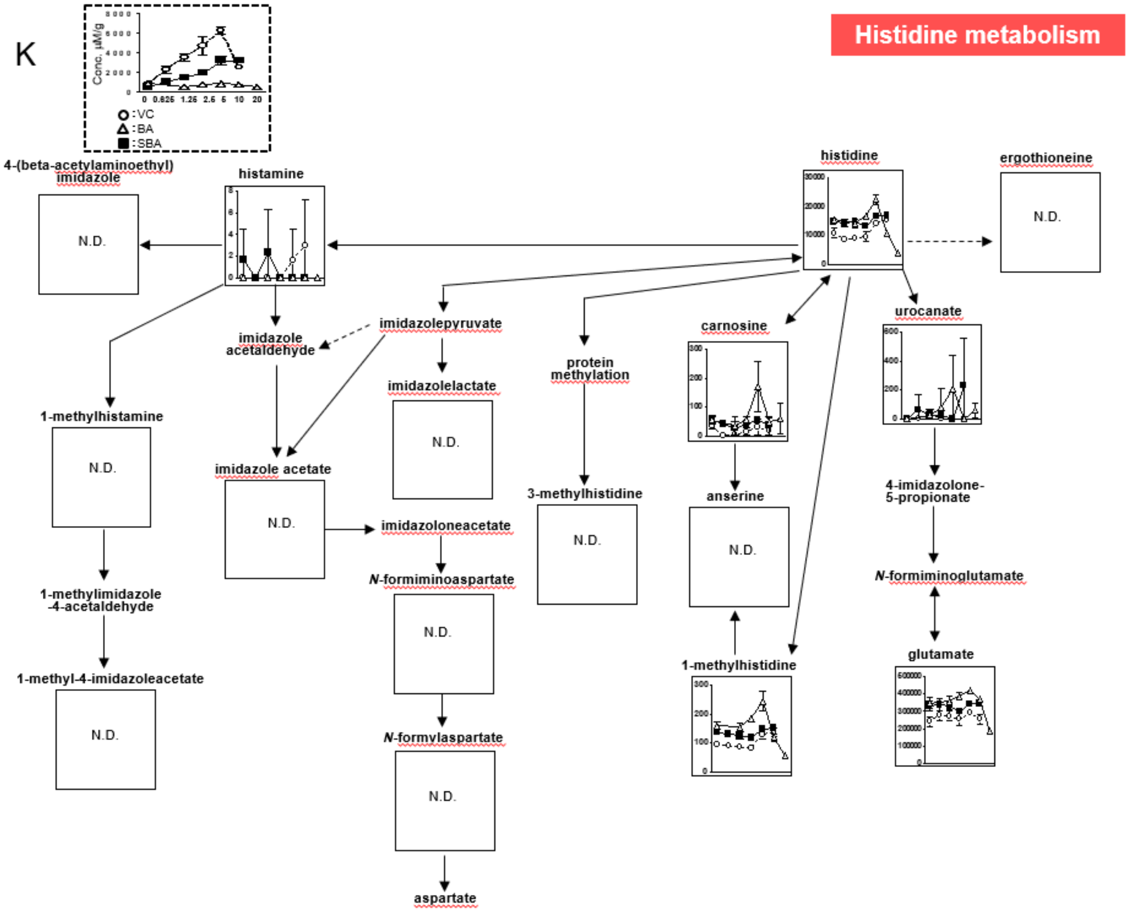
K
Histidine metabolism
Conc. μM/g
0 0.625 1.25 2.5 5 10 20
:VC
:BA
:SBA
4-(beta-acetylaminoethyl) imidazole
N.D.
histamine
histidine
ergothioneine
N.D.
imidazolepyruvate
imidazole acetaldehyde
protein methylation
carnosine
urocanate
1-methylhistamine
N.D.
imidazolelactate
N.D.
imidazole acetate
N.D.
3-methylhistidine
N.D.
anserine
N.D.
4-imidazolone-5-propionate
imidazoloneacetate
N-formiminoaspartate
N.D.
N-formiminoglutamate
1-methylimidazole -4-acetaldehyde
1-methylhistidine
glutamate
1-methyl-4-imidazoleacetate
N.D.
N-formylaspartate
N.D.
aspartate

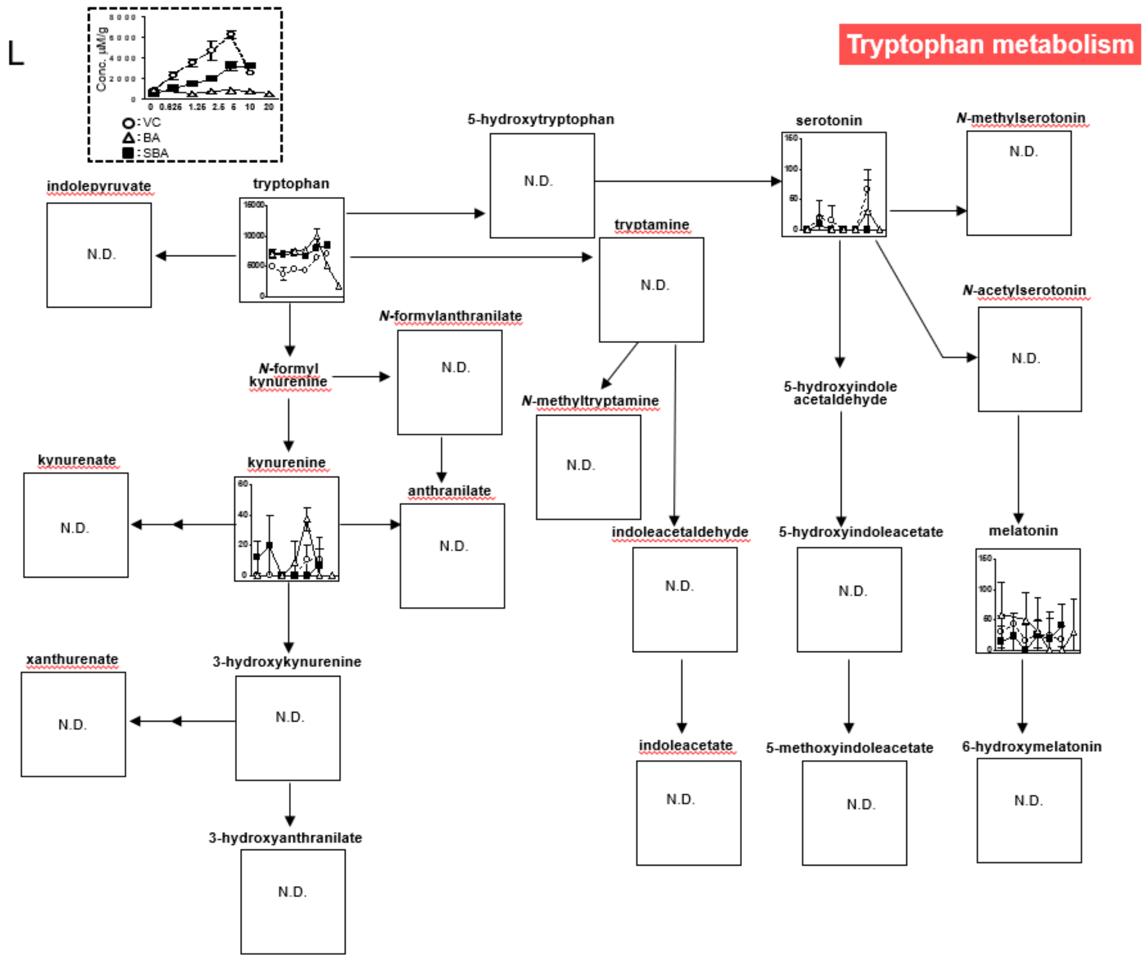
L
Conc. µM/g
0 0.625 1.25 2.5 5 10 20
: VC
: BA
: SBA
Tryptophan metabolism
indolepyruvate
N.D.
tryptophan
5-hydroxytryptophan
N.D.
serotonin
N-methylserotonin
N.D.
tryptamine
N.D.
N-acetylserotonin
N.D.
N-formylanthranilate
N.D.
N-formyl kynurenine
N-methyltryptamine
N.D.
5-hydroxyindole acetaldehyde
kynurenate
N.D.
kynurenine
anthranilate
N.D.
indoleacetaldehyde
N.D.
5-hydroxyindoleacetate
N.D.
melatonin
xanthurenate
N.D.
3-hydroxykynurenine
N.D.
indoleacetate
N.D.
5-methoxyindoleacetate
N.D.
6-hydroxymelatonin
N.D.
3-hydroxyanthranilate
N.D.

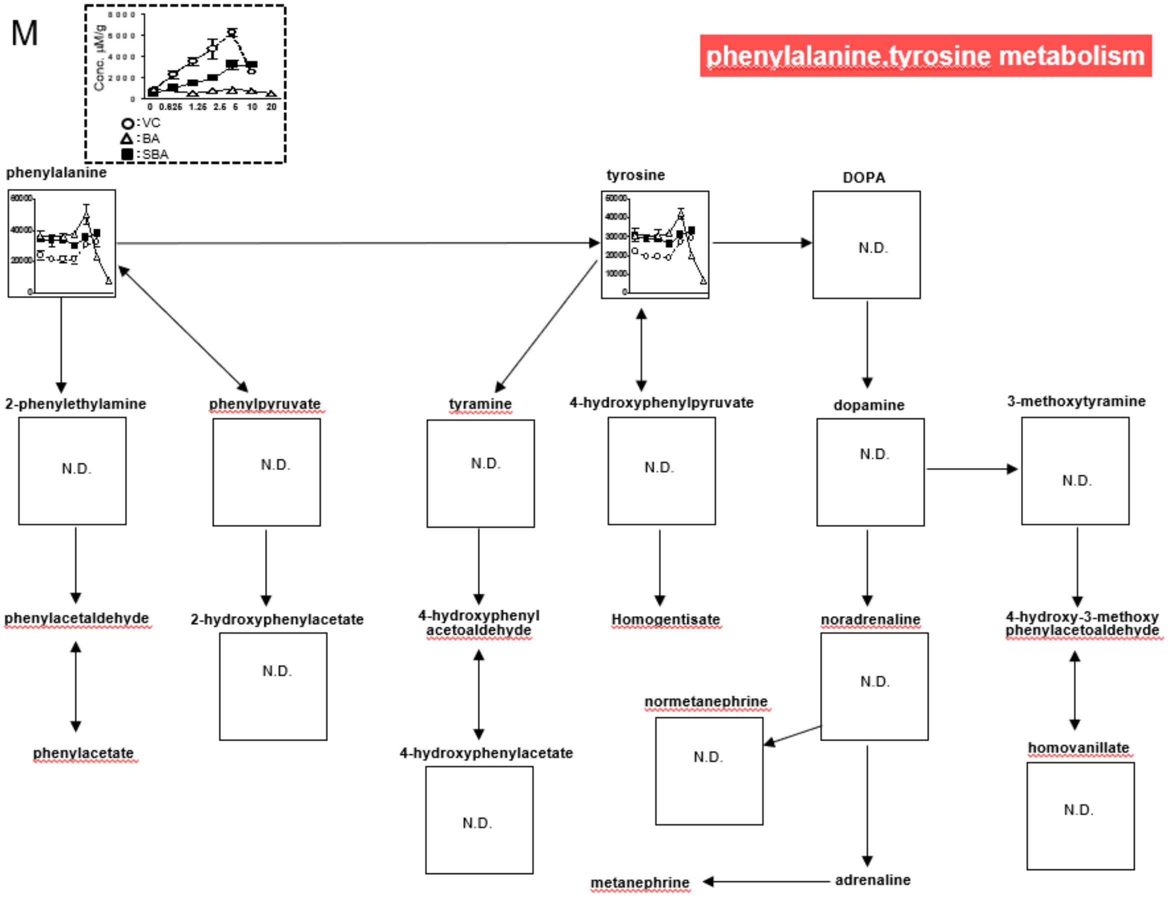
M
Conc. μM/g
0 0.625 1.25 2.5 5 10 20
:VC
:BA
:SBA
phenylalanine.tyrosine metabolism
phenylalanine
tyrosine
DOPA
N.D.
2-phenylethylamine
N.D.
phenylpyruvate
N.D.
tyramine
N.D.
4-hydroxyphenylpyruvate
N.D.
dopamine
N.D.
3-methoxytyramine
N.D.
phenylacetaldehyde
2-hydroxyphenylacetate
N.D.
4-hydroxyphenyl acetoaldehyde
Homogentisate
noradrenaline
N.D.
4-hydroxy-3-methoxy phenylacetoaldehyde
phenylacetate
4-hydroxyphenylacetate
N.D.
normetanephrine
N.D.
homovanillate
N.D.
metanephrine
adrenaline

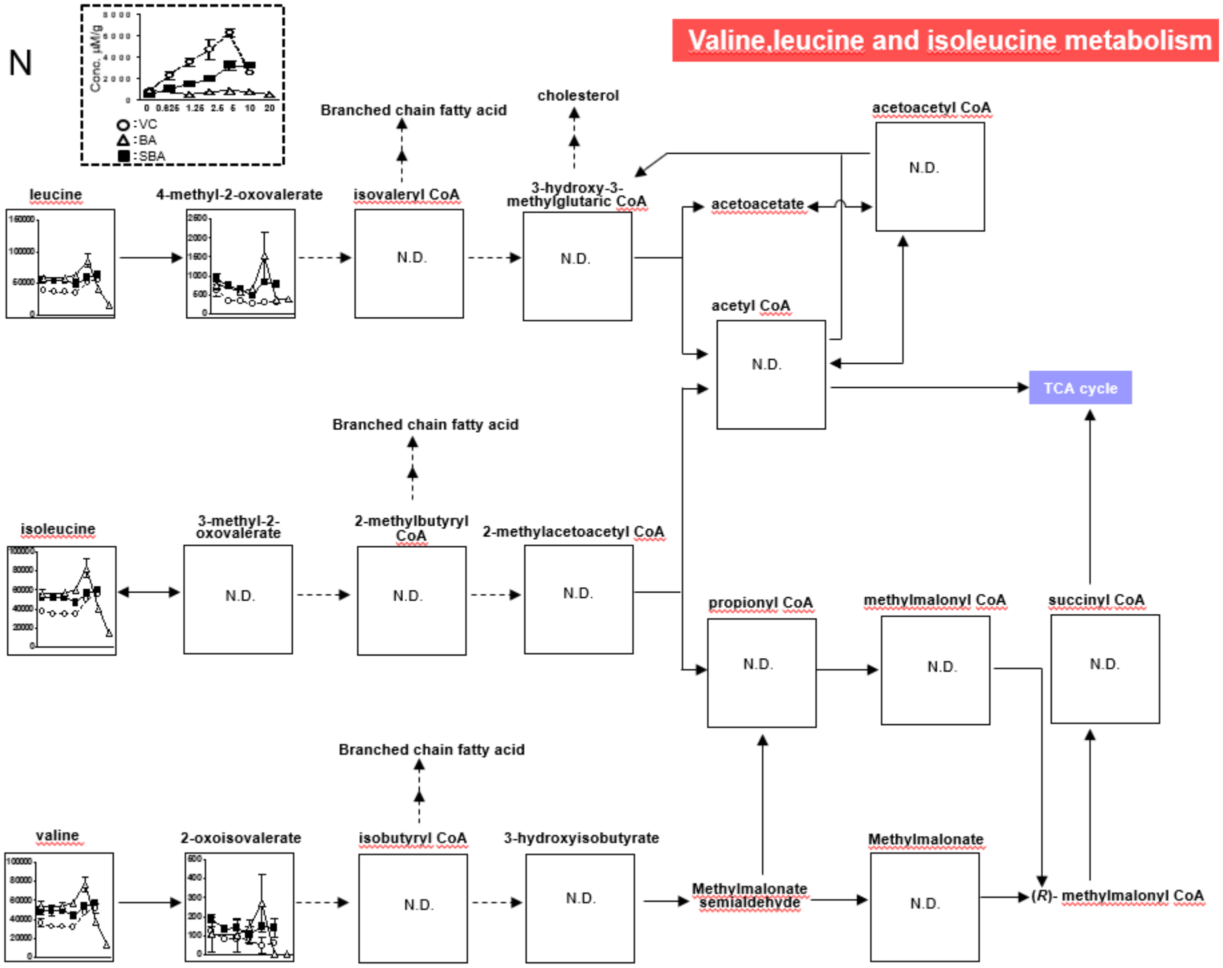
N
Conc. µM/g
:VC
:BA
:SBA
Valine,leucine and isoleucine metabolism
Branched chain fatty acid
cholesterol
acetoacetyl CoA
leucine
4-methyl-2-oxovalerate
isovaleryl CoA
3-hydroxy-3-methylglutaric CoA
acetoacetate
N.D.
acetyl CoA
TCA cycle
isoleucine
3-methyl-2-oxovalerate
2-methylbutyryl CoA
2-methylacetoacetyl CoA
propionyl CoA
methylmalonyl CoA
succinyl CoA
valine
2-oxoisovalerate
isobutyryl CoA
3-hydroxyisobutyrate
Methylmalonate semialdehyde
Methylmalonate
(R)- methylmalonyl CoA

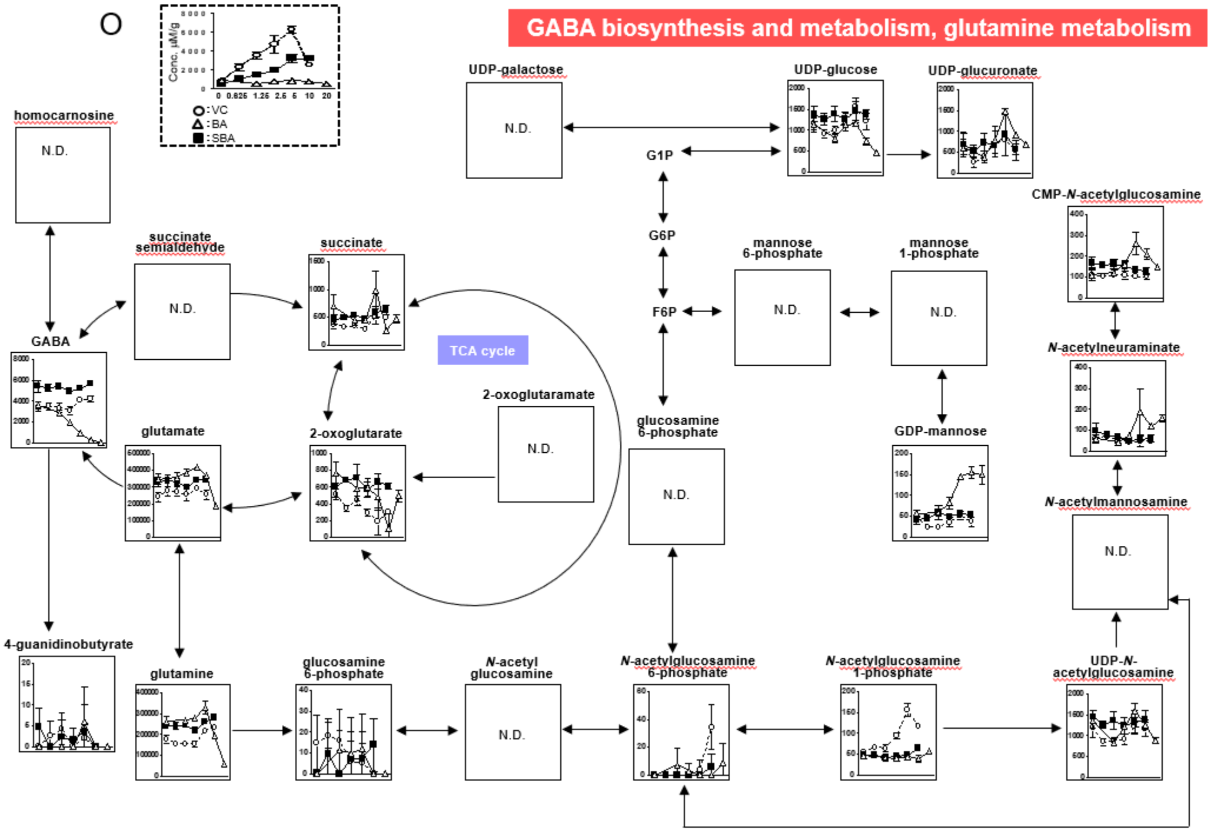

Figure S1. Effect of SBA, BA and SA on Pyrimidine metabolism (**A**), puring metabolism (**B**), glycolysis (**C**), TCA cycle (**D**), pentose phosphate pathway (**E**), arginine, proline metabolism and urea cycle (**F**), glycine, serine and threonine metabolism (**G**), alanine, asparagine and aspartic acid metabolism, β-alanine biosynthesis and metabolism (**H**), lysine metabolisum (**I**), methionine and cysteine metabolism (**G**), histidine metabolism (**K**), tryptophane metabolism (**L**), phenylalanine, tyrosine metabolism (**M**), valine,leucine and isoleucine metabolism (**N**) and GABA biosynthesis and metabolism, glutamine metabolism (**O**) in HSC-2 cells. N.D. not determined.

Table S1. Metabolic changes induced by SBA, BA and SA (A). 171 compounds were detected. Each value represents amol/cell, and can be seen by magnifier.

Compound	Sodium ascorbate 30min treatment															
	0mM			0.625mM		1.25mM			2.5mM			5mM			10mM	
	1	2	3	4	6	7	8	9	10	11	12	13	14	15	16	17
Urea	1985	2231	2842	1923	2160	2065	2118	2600	2439	2240	1898	3509	2306	2544	1932	2780
Gly	45622	56116	60450	44976	54875	44821	52623	55801	40654	52087	53064	57686	68296	66040	63475	76516
Putrescine(1,4-Butanediamine)	165	180	160	62	66	39	80	99	66	183	154	255	229	246	197	102
Sarcosine	67	0	36	35	47	47	30	51	0	47	0	44	0	0	0	0
beta-Ala	1866	2083	2080	1957	2043	1949	2102	2159	1923	1992	1911	2222	2505	2343	2390	2497
Ala	19399	21345	21839	20238	21033	18746	19461	20774	18339	20049	20403	24763	24469	23261	21595	21715
2AB	454	509	578	454	507	416	491	520	444	460	500	632	625	625	646	699
GABA	2970	3630	3863	3270	3731	2948	3399	3788	2666	3330	3369	3808	4276	4156	3990	4410
Choline	384	461	502	377	440	369	426	393	298	396	393	883	841	727	1210	1325
Ser	33790	36325	37596	32994	33210	32045	31575	33860	28913	31283	31842	41248	39247	39214	41108	42725
Diethanolamine	117	111	126	184	0	172	0	109	0	115	98	0	0	116	105	139
Hypotaurine	3542	4333	4518	3919	4334	3899	4343	4711	3467	4129	4564	4529	4856	4730	4621	5160
Carnosine	32	25	46	0	0	15	0	0	0	20	26	58	33	0	36	0
Creatinine	136	184	196	120	163	142	177	180	143	184	159	245	218	162	181	225
Dihydrouracil	29637	27945	30306	47818	45397	44267	37191	31166	34616	26634	25575	35021	36628	29151	39026	48896
Pro	23180	26270	26579	23738	25253	21069	23112	24326	15418	18171	18455	16777	18202	16802	16585	17629
Val	32059	36484	40366	29988	34635	29075	33366	34483	28156	33616	34229	45722	48598	45786	48222	55118
Betaine	167	182	247	154	227	147	197	213	137	185	198	318	228	193	250	276
5-Aminovalerate	168	204	172	152	155	140	155	154	114	151	163	174	219	194	181	207
Thr	51112	71315	75923	51057	63268	49621	61333	67026	46688	60605	62162	74469	82973	79361	78896	91971
Homoserine	0	154	0	0	0	0	0	154	113	169	0	0	206	0	153	200
Nicotinamide	95	43	107	52	45	41	41	41	48	46	42	113	42	32	0	42
Taurine	1489	1508	1711	2147	1983	2108	1937	1655	1630	1542	1474	1940	2095	2036	2189	2489
N-Acetylputrescine	0	0	29	34	0	25	19	27	28	27	25	0	27	25	39	28
Hydroxyproline	881	935	984	925	941	870	888	971	789	897	807	1185	1122	1065	1106	1141
Creatine	31379	34464	33777	27633	28623	27111	28883	29864	29423	30745	31653	73641	80992	75117	109302	110719
Ile	34494	38051	40985	33030	36172	32722	35272	36877	31456	34977	36420	50659	50548	47873	53744	56560
Leu	35878	40354	42390	34474	37098	34919	36773	37521	31815	37175	37143	52873	52763	48629	55278	58569
Asn	3822	1880	1851	1447	1686	1288	1318	1648	1174	1540	1478	1907	2216	1936	1621	2040
Gly-Gly	114	136	151	117	154	121	145	145	85	126	137	169	161	158	172	172
Ornithine	425	327	387	246	301	286	292	339	289	283	309	441	381	357	452	541
Asp	42078	51285	51874	39481	45471	37579	42367	45108	33180	39783	42167	48542	56132	50669	65107	69863
Adenine	89	68	108	69	98	61	71	111	73	94	73	130	117	82	112	133
Hypoxanthine	157	152	260	0	0	0	0	0	55	0	0	262	193	200	160	0
gamma-Guanidinobutyrate	0	0	0	4.6	0	6.1	0	6.5	0	0	0	0	6.1	0	0	0
gamma-Butyrobetaine	74	73	90	73	81	72	81	83	61	73	82	85	96	100	83	98
Gln	156869	187087	191215	149168	165667	145595	160824	162575	136134	163056	163277	216925	223980	214884	226497	242088
Lys	8291	7793	11668	6042	7304	6030	7228	7798	7010	8955	9098	18461	15601	14655	21164	23499
Glu	209292	254695	257348	256446	295576	242626	282080	294241	215151	270619	276062	278209	308513	284710	234878	275289
Met	7618	8304	8572	7275	7758	6798	7367	7539	6148	7433	7507	10169	10150	9763	9939	11346
His	9173	10978	12410	7763	9174	8177	9217	9934	7771	10054	11155	13894	14208	14592	15038	16100
alpha-Aminoadipate	188	272	234	236	303	210	289	300	177	259	307	254	338	328	387	294
Carnitine	254	282	296	273	292	262	276	279	215	240	233	285	265	268	239	263
Methionine sulfoxide	87	92	58	89	68	66	87	101	92	75	58	192	131	93	171	119
Phe	20562	24521	27122	19712	22502	18900	21886	23348	18086	22456	22797	30617	31466	29287	30456	35416
Pyridoxal	20	26	0	0	0	14	25	0	11	20	0	34	20	29	27	36
Pyridoxine	92	75	131	73	69	65	68	90	64	73	69	133	97	85	78	112
3-Methylhistidine	96	91	98	87	96	90	76	88	76	94	81	148	129	113	128	149
Arg	2689	2187	3113	1940	1983	1918	2189	2255	1978	2564	2597	5763	4650	4410	7014	6160
Citrulline	6337	7619	8758	6224	7238	6192	7166	8080	6047	7023	6888	9221	9318	8618	8861	11288
Tyr	21357	21034	23471	19678	18879	19261	18199	19561	17873	18712	19329	28353	26548	25753	28666	30168
Phosphorylcholine	17149	20058	20165	20216	21834	18575	20951	21358	16917	20219	20962	22786	24623	23664	22803	24595
SAH	0	43	32	24	0	0	13	0	0	0	0	0	40	49	28	37
SAM+	876	765	634	600	617	548	557	606	699	743	765	1125	905	1052	1096	982
o-Acetylcarnitine	55	74	73	56	61	54	66	74	79	97	104	140	179	181	249	258
Trp	4683	5044	5390	4450	3035	4261	4507	4697	4044	4412	4598	6584	6534	6008	6849	7280
Cystathionine	810	806	920	746	853	774	807	940	718	787	865	923	1030	921	977	937
Melatonin	45	45	0	39	45	47	0	0	35	44	0	0	70	0	33	0
Cytidine	84	94	98	63	43	67	68	60	77	61	54	170	134	107	130	131
Pyridoxamine 5'-phosphate	25	24	14	20	29	0	20	0	19	0	28	33	0	32	30	43
Glycerophosphorylcholine	1636	1662	1889	1838	1800	1714	1758	1874	1433	1864	2046	2289	2518	2468	3289	3361
Thiamine	289	248	285	193	219	209	224	205	184	259	214	329	289	256	280	300
Adenosine	88	46	93	0	0	0	0	16	40	14	0	292	76	85	136	211
Inosine	218	144	167	0	0	0	0	0	0	0	0	292	186	267	188	297
Glu-Glu	17	19	21	24	13	15	25	19	20	23	20	34	30	27	24	33
Guanosine	113	84	128	0	0	0	0	0	0	0	0	111	78	65	98	151
Ophthalmate	35	0	19	49	41	0	35	45	0	40	29	0	21	0	22	0
5-Methylthioadenosine	15	20	26	14	16	13	13	17	14	15	15	23	21	22	15	27
Glutathione(ox)	6057	7603	8111	16171	7583	6002	7357	7524	6144	7911	5401	29883	11827	7385	9535	17581
Glutathione(red)	28637	32077	28653	17970	30995	29709	31248	33856	25523	29048	36416	1340	31716	35787	32185	21541
Saccharopine	29	0	0	26	31	26	0	35	33	28	0	24	0	0	27	0
Pyruvate	1389	1151	1775	0	0	0	0	0	0	899	656	0	0	0	0	0
Lactate	16248	17214	20235	18080	19589	16004	16078	17492	13131	14321	14836	14659	10032	8109	5406	6975
3-Hydroxybutyrate	47	95	45	0	108	0	36	0	0	0	0	0	35	0	0	39
Fumarate	678	810	649	594	628	550	791	633	436	827	669	674	747	605	361	348
2-Oxoisopentanoate	109	112	151	81	81	68	82	97	84	55	92	0	55	88	53	59
4-Oxopentanoate	105	67	0	0	0	0	0	0	70	0	0	0	0	0	0	0
Hexanoate	41	58	71	36	49	56	43	47	38	36	39	89	58	74	43	53
Succinate	402	279	436	332	304	297	349	362	280	330	256	662	465	411	476	523
2-Hydroxypentanoate	111	103	117	103	108	124	113	118	86	138	133	191	120	151	163	140
5-Oxoproline	3298	2449	4281	2299	2762	2243	2436	2429	1851	2242	1759	4451	2081	1569	1589	2111
4-Methyl-2-oxopentanoate	594	475	800	310	374	331	284	378	219	277	257	344	285	259	252	351
N-Acetyl-beta-alanine	200	183	208	192	282	168	251	207	163	238	236	263	304	223	271	279
Glutarate	134	123	104	124	139	127	163	166	122	149	173	0	157	168	150	138
Malate	2427	2736	2453	2292	2383	2102	2653	2679	1853	2492	2360	2759	2887	2526	2033	2006
Ethanolamine phosphate	68	64	51	54	67	55	47	0	0	0	0	0	0	0	0	0
2-Oxoglutarate	457	500	588	322	373	502	386	426	247	331	295	0	306	263	288	302
2-Hydroxyglutarate	95	71	76	88	93	86	117	117	94	101	133	99	86	106	156	110
trans-Cinnamate	2882	2876	2918	3156	3407	3013	3249	3197	2718	3162	2906	4015	3463	3128	3114	2998
Pelargonate	39	27	39	34	40	28	40	36	31	32	36	61	26	38	47	46
3-Hydroxy-3-methylglutarate	58	55	45	50	65	46	86	89	0	72	0	0	90	79	132	126
Terephthalate	28	23	38	0	32	0	32	0	28	44	0	0	53	38	44	52
PEP	85	133	89	88	101	100	108	81	70	101	99	49	34	32	23	0
Cysteate	0	30	18	17	19	24	0	0	15	0	0	0	0	20	0	0
DHAP	3763	2895	2851	1814	1710	1544	1654	1684	1924	2254	1829	5978	5579	5556	9675	8096
Glycerophosphate	314	383	295	268	244	294	298	261	198	292	260	241	219	199	282	246
Decanoate	31	29	41	0	37	26	27	27	24	27	18	44	38	28	42	23
cis-Aconitate	62	67	63	49	42	47	55	67	46	66	66	158	222	169	423	431
N-Acetylaspartate	497	484	513	541	575	493	603	569	435	493	501	629	692	597	948	766
Carbamoylaspartate	188	146	181	227	195	175	136	203	98	99	99	0	0	0	0	0
O-Phosphoserine	262	205	238	274	273	165	197	244	89	68	109	0	90	0	0	0
3PG	344	470	348	286	312	317	405	350	265	411	373	217	172	145	98	88
Azelate	16	8.6	12	19	22	19	13	14	11	27	41	26	24	16	14	13
N-Acetylglutamate	43	49	34	45	49	37	49	54	36	52	54	58	57	53	63	46
N-Acetylmethionine	68	52	53	65	69	54	56	60	50	69	86	0	77	86	68	59
Isocitrate	56	57	41	34	22	32	18	41	24	25	0	0	32	37	0	0
Citrate	1335	1561	1455	951	1042	901	1101	1017	1015	1295	1122	4317	5040	3757	10769	9483
Dodecanoate	79	70	173	65	65	69	78	67	70	75	48	86	76	60	93	78
Mucate	0	0	0	111	66	103	113	103	131	168	108	2532	298	193	288	499
Pantothenate	402	346	362	374	459	342	457	369	316	417	334	431	482	390	456	416
R5P	431	400	339	332	381	339	407	361	458	567	486	1623	1696	1523	2777	2823
Ru5P	1246	1282	1330	1154	1350	1207	1325	1264	1498	1716	1610	5705	5743	5146	9927	9724
Biotin	789	834	843	895	801	809	886	762	699	803	797	1027	1029	883	974	936
Glucosamine 6-phosphate	0	24	21	23	14	21	15	12	17	0	0	0	16	0	0	0
G6P	643	562	612	792	764	924	967	940	787	825	808	1081	938	752	823	733
F6P	143	140	159	117	102	98	108	91	90	105	82	215	214	197	319	298
G1P	202	192	187	177	173	192	204	200	153	183	141	489	491	428	1293	1074
Sorbitol 6-phosphate	50	45	35	55	37	49	47	42	30	30	30	19	45	37	42	47
2,3-DPG	184	269	104	71	92	73	127	174	89	193	215	67	59	79	0	14
6-Phosphogluconate	866	932	747	2056	2531	3116	3792	3753	3694	5445	5069	6621	6302	5853	2369	2646
S7P	112	145	101	129	120	123	112	126	139	165	147	328	286	291	423	395
Argininosuccinate	69	78	82	62	57	68	45	65	56	38	54	94	76	72	54	79
N-Acetylglucosamine 1-phosphate	51	61	52	70	64	74	62	57	87	101	98	174	147	148	119	117
dCMP	16	0	8.7	0	0	0	0	0	0	0	0	0	9.1	0	23	0
N-Acetylneuraminate	62	50	68	76	68	40	53	69	34	45	44	78	38	30	49	45
Ribulose 1,5-diphosphate	60	64	51	41	67	65	57	60	64	76	96	162	155	142	183	158
dTMP	18	16	17	11	11	13	0	17	12	0	10	28	40	40	58	62
CMP	224	92	135	26	25	13	15	26	50	30	26	392	373	340	427	367
UMP	900	433	612	205	178	184	191	195	239	239	192	1513	1448	1306	1695	1745
dAMP	0	0	0	0	0	0	0	0	0	0	0	0	16	20	23	30
F1,6P	7739	6450	5566	2422	2762	2387	2836	3023	3365	4093	3394	16832	17748	16202	31779	24536
AMP	1828	577	1090	151	146	114	100	105	358	230	167	2822	2882	2669	3891	3589
IMP	156	70	94	32	24	12	0	26	57	46	25	1226	1276	1179	1828	1641
GMP	205	121	131	58	48	55	50	73	70	55	63	544	507	485	936	889
XMP	0	0	0	0	0	0	0	0	0	0	0	17	0	10	16	14
NADPH	171	173	130	67	98	86	178	199	117	237	312	215	200	316	226	139
CoA	75	119	74	0	23	0	55	49	46	109	148	0	95	159	97	73
dCDP	17	0	12	0	0	0	0	0	14	0	0	0	17	0	0	0
PRPP	84	63	0	28	0	0	44	36	49	58	74	173	105	118	74	0
dTDP	26	16	24	0	0	0	0	0	0	10	0	35	30	30	32	21
CDP	350	188	262	42	45	52	46	39	95	91	74	356	298	275	151	142
UDP	854	558	637	99	109	108	113	97	215	178	152	791	698	662	515	377
dADP	19	18	0	0	0	0	0	0	0	0	0	25	20	19	19	0
Trehalose 6-phosphate	0	0	0	32	78	98	149	86	171	211	140	2523	458	277	595	535
Adenosine 5'-phosphosulfate	0	16	12	0	0	0	0	0	0	11	0	31	0	0	0	0
ADP	3319	2325	2558	582	641	673	736	744	1019	1136	983	3197	3052	2798	2085	1886
GDP	751	485	429	89	124	124	129	161	209	216	206	922	812	842	671	506
Adenylosuccinate	20	29	0	0	0	0	0	0	0	0	0	52	61	75	43	50
dCTP	18	34	13	16	0	19	22	33	22	32	30	19	19	33	0	0
dTTP	44	88	37	32	50	51	62	49	50	90	81	63	43	45	28	18
CTP	666	1108	493	526	698	632	829	948	703	1277	1337	759	744	776	180	184
UTP	1460	3213	1239	1019	1681	1436	2395	2285	1477	3332	2973	1403	1772	1596	405	316
dATP	25	66	33	0	40	33	43	35	39	52	74	0	35	49	0	0
ATP	5433	11375	4693	3854	5818	5143	8213	7726	5987	12797	12371	6575	6477	6566	1314	1247
ITP	500	1008	457	388	494	490	752	717	540	1167	1169	639	598	658	109	116
GTP	2124	3580	1109	1169	1857	1442	2472	2526	2077	3961	3951	2500	2305	2467	836	704
CDP-choline	48	42	22	0	0	0	0	0	0	0	0	148	181	180	261	255
UDP-glucose	1151	1389	959	845	974	901	1085	1026	963	1358	1274	1694	1555	1439	1364	1064
UDP-glucuronate	618	1018	444	167	339	234	443	499	353	942	840	760	765	857	718	370
ADP-glucose	42	61	37	31	30	30	39	38	19	42	46	46	42	55	57	30
GDP-mannose	44	67	34	23	26	17	26	24	23	46	39	43	48	63	45	28
UDP-N-acetylglucosamine	1161	1434	980	793	902	786	881	943	775	1003	958	1340	1225	1080	1317	1042
CMP-N-acetylneuraminate	98	135	90	109	100	110	107	115	85	127	112	107	110	89	111	91
NAD+	1185	1263	1223	1040	1097	964	989	979	447	466	489	233	259	239	170	168
NADH	441	509	278	387	327	278	490	530	189	340	345	0	163	196	0	147
NADP+	34	67	25	0	0	0	0	0	18	36	37	28	32	36	30	37
FAD	0	51	30	0	0	0	0	0	0	0	53	0	0	0	43	0
Glu+ threo-beta-methylaspartate	58070	57223	57414	64638	66453	63474	63255	63620	55335	60642	60400	78850	65650	60898	62058	57434
Threonate	390	419	481	712	1256	816	1331	1085	900	1599	1056	15933	2455	1416	2288	3259
G3P	454	220	314	474	470	362	243	264	653	267	412	1876	1489	1408	2381	1852
2PG	76	53	45	59	78	72	60	82	60	63	77	46	47	39	0	0

Compound	Sodium 5,6-benzylidene-L-ascorbate (SBA) 60min treatment																	
	0mM			0.625mM			1.25mM			2.5mM			5mM			10mM		
	19	20	21	22	23	24	25	26	27	28	29	30	31	32	33	34	35	36
Urea	2926	3197	2944	4298	3466	3342	3032	3071	2852	3016	2554	2385	4162	3922	2949	3048	3401	4188
Gly	56280	69869	66661	57282	71361	67353	62058	69181	68857	60421	65400	59542	72611	81137	77249	81581	88945	90183
Putrescine(1,4-Butanediamine)	155	285	178	88	115	98	160	158	97	78	102	148	217	258	108	90	127	142
Sarcosine	0	0	40	53	54	83	0	58	0	71	0	42	48	72	83	55	72	0
beta-Ala	2534	2584	2611	2503	2738	2656	2526	2773	2514	2575	2439	2216	2601	2565	2502	2962	2733	2834
Ala	27782	28803	28625	28241	31321	29451	28247	29835	27340	27829	27181	24940	31317	31995	30767	32882	31547	33645
2AB	682	707	704	650	735	708	669	779	728	691	631	640	777	828	806	842	890	907
GABA	4845	5944	5621	4919	5630	5252	5362	5488	5454	5058	5134	4695	5276	5368	5106	5673	5601	5849
Choline	732	824	712	732	820	751	761	823	730	623	642	590	719	709	707	835	837	800
Ser	49386	51368	49521	51565	53780	50572	50169	53263	50985	49735	47956	44134	56721	57763	54971	58176	59616	60351
Diethanolamine	0	0	0	223	273	103	137	0	0	0	102	358	212	0	0	0	193	151
Hypotaurine	4994	5262	5451	5072	5454	5583	4966	5672	5545	5150	5047	4695	5520	5426	5347	5611	5801	6106
Carnosine	50	67	44	42	46	40	39	40	0	37	39	48	50	62	53	49	61	0
Creatinine	231	260	229	199	272	226	210	246	215	204	212	189	260	276	213	268	288	253
Dihydrouracil	42924	47617	47035	48998	57835	59719	45846	40653	45692	56729	40466	34666	59882	36686	47987	57629	49435	50371
Pro	28755	30362	30610	26214	31230	28240	25758	27689	26038	23721	23537	21695	23520	25275	24545	23292	23245	24076
Val	44824	53323	50163	42413	53866	50128	47145	51819	49125	42865	45331	42880	49915	55822	53695	54524	59345	57319
Betaine	228	307	252	204	280	262	271	308	255	202	270	215	233	362	281	272	366	386
5-Aminovalerate	508	549	573	436	632	543	497	596	525	501	561	464	500	501	568	545	566	575
Thr	79653	97234	89510	79660	100369	90349	84959	96963	90162	84380	88475	79197	95882	107305	98279	99742	110375	105030
Homoserine	0	0	0	0	0	124	0	270	0	0	232	192	202	292	0	191	286	252
Nicotinamide	146	131	92	103	121	88	126	60	62	51	57	59	145	146	104	134	134	132
Taurine	2405	2611	2449	2803	2898	2999	2620	2164	2454	2643	2273	1812	2213	2357	2635	3019	2458	3041
N-Acetylputrescine	21	30	30	25	32	23	31	13	24	26	24	31	26	27	30	30	23	38
Hydroxyproline	1197	1178	1132	1149	1209	1212	1209	1242	1211	1217	1081	972	1318	1423	1310	1418	1433	1503
Creatine	43926	49447	49335	48047	53827	50402	49626	52778	50451	54040	51555	48301	59092	62333	58086	67166	63664	67301
Ile	52087	53689	51526	47811	54998	52106	51989	53979	51190	48505	47292	44716	56409	57724	55201	59596	59660	60123
Leu	53138	55883	53628	50040	58339	53948	53707	55339	52776	50829	49760	46504	57206	60519	57657	62116	63494	62350
Asn	1832	2024	2032	1697	1938	1723	1713	1753	1872	1761	1727	1771	1906	2086	1902	2145	2227	2493
Gly-Gly	151	153	159	134	158	161	130	168	155	156	149	143	160	198	164	159	183	207
Ornithine	478	514	588	1612	486	515	522	453	420	350	376	360	475	549	390	416	633	3710
Asp	72153	81068	79599	73498	84350	79890	71566	77939	78539	72078	68391	68342	77406	85595	81692	82001	82391	84781
Adenine	117	114	115	97	165	128	133	152	129	95	149	131	144	166	98	108	161	121
Hypoxanthine	242	447	471	212	235	251	0	0	0	171	0	139	147	176	135	0	0	291
gamma-Guanidinobutyrate	5.1	9.1	0	0	0	0	0	7.2	0	0	0	4.8	0	11	0	0	0	0
gamma-Butyrobetaine	95	111	108	101	123	113	100	119	108	99	99	90	110	121	105	120	125	115
Gln	229641	251386	240479	223964	263183	241600	238325	251949	244021	224407	224935	213957	260145	270165	256735	283187	286625	280829
Lys	14657	14785	11864	11760	14403	12449	14072	12791	11927	9585	12039	10932	16154	18547	14435	15250	19548	19094
Glu	300458	355513	334709	299538	364010	351669	307122	349911	337625	298717	300008	299101	331061	355566	344010	329017	352096	362159
Met	11963	11128	11013	11020	11254	10421	11755	11476	10499	10786	9998	9144	12367	12482	11232	12827	12482	11962
His	14009	15695	14883	13035	15602	14568	13959	15995	14631	12905	13927	12949	16742	17902	15602	16147	17820	16715
alpha-Aminoadipate	267	323	351	288	360	327	286	330	323	300	280	259	312	339	300	283	295	329
Carnitine	392	423	388	350	416	391	373	399	351	354	351	315	393	383	381	376	396	369
Methionine sulfoxide	121	149	128	114	147	114	114	122	129	104	130	129	44	140	139	147	158	259
Phe	32002	36046	34523	29495	36968	34318	32397	34615	33227	30402	31149	28768	33953	37767	35590	36567	38318	39212
Pyridoxal	30	28	31	28	28	29	24	25	26	18	23	24	21	35	36	30	0	28
Pyridoxine	145	133	134	116	163	133	136	132	135	99	107	114	125	162	136	129	163	152
3-Methylhistidine	142	140	129	130	128	133	139	113	127	120	118	113	154	139	149	144	148	165
Arg	5217	4536	3248	3859	4490	3799	4751	4332	3864	3333	3822	3524	5444	6073	4481	5063	6049	2991
Citrulline	6705	7483	7420	6280	7630	6919	6560	7157	6617	5924	6218	5883	6754	7214	6613	6313	6853	6708
Tyr	30153	30094	30331	27215	31400	29101	28036	29726	28301	26427	27061	24578	29671	32367	30810	32051	33349	34561
Phosphorylcholine	22363	24433	23807	23350	26840	24792	22976	25158	25016	22006	21911	20367	23211	24664	24460	23460	24430	25593
SAH	41	40	65	42	0	32	0	0	0	62	43	35	0	0	44	36	0	0
SAM+	1151	1014	1000	1002	1025	1131	1198	1142	1197	1033	991	1224	1454	1192	1043	940	1104	1125
o-Acetylcarnitine	77	85	91	94	114	98	104	124	109	119	124	104	112	112	109	112	115	112
Trp	6909	7040	6887	6476	7432	7195	7029	7379	7064	6939	6914	6259	8016	8052	7711	8397	8470	8398
Cystathionine	990	1025	1015	1010	1151	1075	1043	1090	1018	912	987	923	1026	1056	989	1009	1026	1070
Melatonin	0	0	43	67	0	0	0	0	0	36	0	40	0	57	0	64	0	58
Cytidine	152	182	126	105	119	121	111	129	105	101	136	141	139	164	133	110	146	130
Pyridoxamine 5'-phosphate	32	0	26	26	30	15	0	33	0	21	28	20	31	20	26	24	33	33
Glycerophosphorylcholine	2105	2715	2812	2268	2731	2788	2342	2720	2840	2247	2376	2320	2727	3241	3125	3069	3606	3555
Thiamine	410	381	354	339	360	275	388	324	299	302	305	288	430	384	342	396	400	392
Adenosine	160	155	164	28	61	69	47	30	46	39	59	72	64	40	32	23	58	42
Inosine	334	507	500	147	239	185	130	0	0	136	126	0	0	0	0	0	0	0
Glu-Glu	24	22	24	21	29	28	25	22	25	21	27	23	20	29	21	23	34	30
Guanosine	206	252	197	49	65	103	50	0	58	57	98	85	81	52	33	0	0	0
Ophthalmate	25	58	33	61	49	0	54	58	63	69	64	56	76	73	66	132	115	80
5-Methylthioadenosine	23	33	25	25	31	19	29	29	26	18	29	29	27	30	21	22	28	24
Glutathione(ox)	7091	9978	6481	6232	10968	6528	7088	7239	5951	10521	18137	5399	16133	12240	9269	23371	18211	16560
Glutathione(red)	42316	43983	48195	45301	47883	50017	47740	47061	50178	43558	24641	42298	38273	37669	40203	22477	25804	29890
Saccharopine	80	71	66	56	69	70	66	64	53	68	63	55	61	0	64	57	62	60
Pyruvate	3049	2262	1882	2697	2322	2381	2513	2036	2223	2191	1728	1388	2639	2339	2475	2871	2490	2535
Lactate	21766	22185	21003	28423	24884	22006	22313	19101	20407	17944	16138	13672	30348	24677	24525	27832	24822	27788
3-Hydroxybutyrate	0	99	71	111	82	63	0	74	85	0	0	0	0	0	70	0	116	82
Fumarate	770	1107	796	773	812	839	897	1028	805	870	1039	767	1006	1287	959	1126	1368	1132
2-Oxoisopentanoate	173	211	178	122	150	129	154	174	96	142	117	58	168	132	140	109	117	198
4-Oxopentanoate	113	86	44	0	87	69	122	87	94	56	90	77	129	78	87	0	84	90
Hexanoate	66	66	50	68	55	54	48	59	41	43	53	48	42	56	55	66	52	69
Succinate	503	524	374	489	507	481	550	568	504	435	530	419	644	658	478	618	729	571
2-Hydroxypentanoate	156	120	150	113	118	95	126	97	127	114	110	71	214	123	132	115	154	159
5-Oxoproline	5369	4632	3530	4958	4053	4152	4826	3417	3527	2983	3088	2548	4889	4709	3922	4649	5260	4521
4-Methyl-2-oxopentanoate	997	990	762	790	754	656	734	582	569	546	506	358	859	853	765	820	728	780
N-Acetyl-beta-alanine	239	287	192	218	204	234	215	276	194	209	228	185	283	264	212	267	273	233
Glutarate	191	174	155	173	139	146	211	150	153	0	187	106	169	158	156	176	180	90
Malate	2947	3568	3327	3320	3168	3246	3204	3588	3590	3320	3332	3386	3910	4115	3404	4203	4400	3656
Ethanolamine phosphate	0	34	50	76	62	72	0	48	49	70	0	64	41	0	63	80	57	62
2-Oxoglutarate	470	734	613	724	688	646	695	888	553	671	573	488	745	687	573	658	577	609
2-Hydroxyglutarate	166	183	150	119	104	105	202	124	153	128	139	140	136	128	117	0	155	128
trans-Cinnamate	3770	3792	3601	3931	3777	3732	3650	3620	3610	3667	3260	3153	3923	3919	3734	3980	3539	4083
Pelargonate	37	49	38	45	37	47	41	56	36	56	47	53	42	42	46	54	70	48
3-Hydroxy-3-methylglutarate	71	81	74	0	68	79	83	65	60	65	83	80	0	93	72	133	124	96
Terephthalate	0	37	29	39	34	42	0	32	45	0	41	31	29	45	36	0	55	30
PEP	78	87	72	111	117	95	144	175	137	167	140	147	375	321	289	412	431	471
Cysteate	0	0	21	0	16	0	0	0	0	0	0	0	22	13	0	0	20	21
DHAP	3814	4167	4501	3356	4050	4642	3225	3270	3344	3938	3244	3059	3859	3305	3803	3657	3740	4490
Glycerophosphate	458	469	449	457	499	463	442	426	468	466	486	380	583	494	479	545	565	530
Decanoate	30	32	25	27	28	33	0	24	29	28	0	34	39	41	21	37	26	28
cis-Aconitate	76	96	56	126	96	99	113	146	120	129	128	77	144	142	115	139	123	125
N-Acetylaspartate	563	677	547	596	537	584	635	639	626	557	599	507	603	631	570	643	675	645
Carbamoylaspartate	182	137	106	132	82	137	140	113	142	170	148	139	133	91	113	71	131	136
O-Phosphoserine	205	157	151	291	255	291	376	350	344	546	461	384	729	677	683	592	699	733
3PG	411	430	300	403	391	334	509	530	358	523	476	430	967	741	655	952	875	891
Azelate	0	18	10	26	20	30	16	14	11	16	25	14	48	23	12	22	17	20
N-Acetylglutamate	62	42	39	46	38	42	43	45	41	49	41	37	56	39	32	54	48	52
N-Acetylmethionine	75	71	68	64	47	52	65	61	54	46	53	51	51	63	54	58	66	67
Isocitrate	59	37	40	57	66	48	82	79	65	74	71	75	88	63	67	98	63	71
Citrate	1631	1706	1441	1841	1683	1643	2133	2304	2002	2221	2131	1914	2427	2476	1998	2415	2182	2062
Dodecanoate	80	92	205	95	67	75	80	68	173	94	68	166	82	64	97	92	90	93
Mucate	0	0	0	33	40	28	37	27	34	31	61	31	73	78	68	128	105	105
Pantothenate	506	475	415	414	428	437	396	422	370	354	390	337	451	463	392	426	455	410
R5P	366	339	336	253	324	328	313	333	314	384	394	320	471	449	418	461	434	403
Ru5P	1219	1195	1138	1097	1068	1079	1062	1059	1053	1361	1220	995	1443	1466	1349	1503	1514	1447
Biotin	956	941	880	1013	1082	1034	888	846	948	930	818	815	1036	871	1000	941	863	1109
Glucosamine 6-phosphate	0	0	0	29	0	0	0	0	0	0	0	22	0	0	22	17	0	25
G6P	836	744	751	1052	980	924	1145	1025	1083	946	883	753	1500	1376	1295	1356	1313	1381
F6P	196	186	193	219	215	174	195	180	165	156	116	120	236	191	219	219	169	222
G1P	276	259	277	306	284	268	304	273	261	243	212	220	329	337	283	323	315	333
Sorbitol 6-phosphate	42	47	35	38	41	27	38	27	35	57	34	34	64	37	42	50	37	48
2,3-DPG	149	173	79	230	121	112	203	226	135	175	202	224	482	315	201	256	223	224
6-Phosphogluconate	565	512	486	1166	944	950	1523	1606	1293	1870	1993	1911	3603	3179	2719	3171	3168	3222
S7P	133	115	110	122	107	115	140	131	115	117	130	118	174	161	129	188	176	155
Argininosuccinate	113	102	105	113	89	121	84	111	107	120	98	87	137	143	115	122	130	97
N-Acetylglucosamine 1-phosphate	49	51	40	51	44	50	44	34	44	45	48	38	46	52	48	58	65	69
dCMP	20	14	8.5	0	0	0	0	0	12	0	12	0	0	0	0	0	0	0
N-Acetylneuraminate	140	79	72	88	74	72	72	66	61	61	49	38	60	52	53	82	46	59
Ribulose 1,5-diphosphate	61	34	54	65	67	52	87	54	83	55	69	83	105	89	84	103	88	76
dTMP	27	28	25	0	21	17	23	16	23	28	8.6	18	24	0	16	0	0	14
CMP	377	316	237	110	133	131	114	70	53	125	126	110	67	73	55	49	49	58
UMP	1550	1393	1121	565	668	666	575	428	375	636	606	487	459	435	367	335	306	312
dAMP	18	13	14	0	0	0	0	0	0	0	0	0	0	0	0	0	0	0
F1,6P	10016	10819	8463	6024	6206	5864	6712	6834	5070	6681	6804	6339	7378	6508	5398	5729	5684	5614
AMP	3091	2626	2182	797	989	1081	915	554	461	913	935	780	523	483	406	380	318	335
IMP	259	373	294	129	133	118	88	44	48	110	102	88	77	40	34	90	39	53
GMP	394	322	248	189	214	186	189	124	108	198	120	145	137	112	119	108	97	79
XMP	0	0	0	0	0	0	0	0	0	0	0	0	0	0	0	0	0	0
NADPH	189	172	96	259	162	88	159	247	121	139	198	174	373	217	254	203	139	139
CoA	80	132	66	72	81	55	60	133	67	59	41	103	94	94	71	96	0	84
dCDP	22	0	21	22	6.2	0	23	14	6.1	16	14	9.1	0	0	0	0	0	0
PRPP	86	44	0	70	23	37	93	58	0	82	0	0	123	65	0	0	47	52
dTDP	36	31	26	0	12	23	0	20	0	0	21	0	0	20	0	0	0	0
CDP	466	385	342	228	235	232	288	216	173	255	286	233	234	209	147	163	155	156
UDP	1230	1240	918	612	684	621	779	609	436	686	651	610	558	500	372	362	462	350
dADP	30	28	17	22	12	0	20	0	6.2	20	9.6	16	0	0	10	0	0	0
Trehalose 6-phosphate	0	0	0	0	0	0	24	15	0	26	12	13	65	63	39	67	53	49
Adenosine 5'-phosphosulfate	0	0	0	0	0	0	0	13	0	0	0	0	0	0	13	0	0	0
ADP	4462	4276	3175	2440	2580	2340	3166	2627	1755	2642	2870	2761	2858	2559	1739	1765	2015	1622
GDP	972	873	574	480	498	495	622	491	353	458	565	545	558	444	286	261	340	273
Adenylosuccinate	53	42	29	0	24	16	0	0	0	0	14	22	0	0	0	0	0	0
dCTP	20	18	12	39	12	23	30	27	11	18	26	18	53	34	17	37	30	35
dTTP	46	53	30	50	40	31	65	61	59	64	58	45	80	59	71	68	69	57
CTP	573	532	410	833	658	578	1044	925	676	790	709	784	1629	1054	803	1044	949	906
UTP	1380	1597	1122	2122	1581	1388	2350	2571	1760	1985	1953	1958	3648	2991	1954	2517	2642	2149
dATP	0	47	30	0	24	0	0	39	23	0	0	37	68	46	41	0	33	0
ATP	4644	5004	3955	7245	5408	4312	8008	9037	6332	6666	6454	7293	14162	10256	7037	9488	9494	7489
ITP	429	486	356	698	499	415	784	801	573	610	618	689	1280	940	675	905	842	734
GTP	2010	1752	995	2236	1865	1828	2872	3115	2116	1968	2286	2411	5378	3350	1907	2598	2769	1998
CDP-choline	60	95	87	0	44	51	0	0	0	78	48	0	0	0	0	0	0	60
UDP-glucose	1542	1438	1194	1417	1272	1123	1575	1396	1223	1461	1178	1186	1800	1410	1212	1495	1366	1295
UDP-glucuronate	736	917	414	690	472	432	816	908	489	592	687	738	1475	866	462	629	652	429
ADP-glucose	65	57	32	46	41	45	49	51	51	49	49	52	59	55	45	34	54	43
GDP-mannose	46	45	35	51	41	40	78	44	53	44	52	52	62	56	52	48	60	52
UDP-N-acetylglucosamine	1611	1479	1285	1341	1309	1173	1600	1326	1176	1460	1143	1122	1619	1267	1069	1631	1309	1205
CMP-N-acetylneuraminate	167	196	145	174	155	145	189	159	141	177	142	133	151	124	125	149	118	116
NAD+	1673	1516	1347	1576	1660	1296	1496	1310	1279	1364	1207	1161	1561	1217	1223	1158	1033	1053
NADH	[illegible]	[illegible]	[illegible]	[illegible]	[illegible]	[illegible]	[illegible]	[illegible]	[illegible]	[illegible]	[illegible]	[illegible]	[illegible]	[illegible]	[illegible]	[illegible]	[illegible]	[illegible]
NADP+	[illegible]	[illegible]	[illegible]	[illegible]	[illegible]	[illegible]	[illegible]	[illegible]	[illegible]	[illegible]	[illegible]	[illegible]	[illegible]	[illegible]	[illegible]	[illegible]	[illegible]	[illegible]
FAD	[illegible]	[illegible]	[illegible]	[illegible]	[illegible]	[illegible]	[illegible]	[illegible]	[illegible]	[illegible]	[illegible]	[illegible]	[illegible]	[illegible]	[illegible]	[illegible]	[illegible]	[illegible]
Glu+ threo-beta-methylaspartate	[illegible]	[illegible]	[illegible]	[illegible]	[illegible]	[illegible]	[illegible]	[illegible]	[illegible]	[illegible]	[illegible]	[illegible]	[illegible]	[illegible]	[illegible]	[illegible]	[illegible]	[illegible]
Threonate	[illegible]	[illegible]	[illegible]	[illegible]	[illegible]	[illegible]	[illegible]	[illegible]	[illegible]	[illegible]	[illegible]	[illegible]	[illegible]	[illegible]	[illegible]	[illegible]	[illegible]	[illegible]
G3P	[illegible]	[illegible]	[illegible]	[illegible]	[illegible]	[illegible]	[illegible]	[illegible]	[illegible]	[illegible]	[illegible]	[illegible]	[illegible]	[illegible]	[illegible]	[illegible]	[illegible]	[illegible]
2PG	[illegible]	[illegible]	[illegible]	[illegible]	[illegible]	[illegible]	[illegible]	[illegible]	[illegible]	[illegible]	[illegible]	[illegible]	[illegible]	[illegible]	[illegible]	[illegible]	[illegible]	[illegible]

Compound	Benzaldehyde (BA) 90min treatment																
	0mM			1.25mM			2.5mM			5mM		10mM			20mM		
	37	38	39	40	41	42	43	44	45	46	47	49	50	51	52	53	54
Urea	3726	4120	3839	3617	3426	4243	4141	4401	4206	9795	8091	4341	3569	3983	2895	3084	3409
Gly	58570	67845	73197	67816	76429	80770	79149	83584	85749	92647	101641	57639	59925	61919	13236	14111	14316
Putrescine(1,4-Butanediamine)	153	173	119	74	99	118	82	135	65	240	291	105	65	83	103	133	66
Sarcosine	0	0	90	79	61	0	80	0	0	51	77	90	0	77	0	0	0
beta-Ala	2382	2187	2871	2195	2240	2635	2063	1623	1928	1306	1350	481	359	432	146	199	162
Ala	28380	29193	32120	27257	29827	31780	31181	29346	31705	33294	34230	19426	19036	19730	5060	4712	5009
2AB	782	868	979	805	914	925	873	880	932	1270	1054	552	551	599	227	175	249
GABA	3258	3676	3925	2505	3114	2989	2007	1767	2058	860	937	212	223	275	0	0	134
Choline	677	744	741	789	872	862	1075	985	963	1786	1458	1314	1117	1296	1235	1246	1132
Ser	45603	50455	55853	47473	52663	52818	55571	52176	54437	62920	65001	35466	36189	37011	10362	10623	11099
Diethanolamine	460	395	305	0	248	139	304	199	280	181	204	183	269	305	324	0	332
Hypotaurine	5428	5729	7401	6022	6872	6981	6104	5588	6482	5537	6330	5007	4543	5313	1729	1557	1628
Carnosine	41	56	70	59	49	0	67	56	42	234	110	41	67	41	0	104	73
Creatinine	254	344	271	242	274	273	298	315	271	646	436	251	213	274	165	138	194
Dihydrouracil	62354	64418	59626	80384	67841	65603	68467	46158	52945	55769	49518	51203	54844	48632	54361	59937	55984
Pro	28067	29933	33919	28126	30264	31709	33129	36886	37738	47292	47409	39663	44441	41176	10186	9831	10812
Val	47337	55467	58694	49267	55295	57600	56547	57942	57720	83043	71872	36860	37881	38206	12780	14600	13557
Betaine	366	428	378	304	433	413	354	432	426	590	553	278	236	327	182	267	247
5-Aminovalerate	287	335	338	212	228	316	143	143	280	0	95	0	0	0	0	0	0
Thr	90536	104913	111665	94740	102858	105677	100579	104574	106431	126548	122310	66583	70798	68500	20998	22541	20986
Homoserine	236	356	0	249	324	0	0	379	0	367	394	400	334	294	267	277	300
Nicotinamide	80	109	71	55	42	39	115	80	56	666	319	55	61	67	107	107	102
Taurine	2247	2565	2919	3296	2850	2886	2862	1836	2376	2315	2263	2032	1639	2065	1001	1206	977
N-Acetylputrescine	0	37	0	0	0	0	0	0	0	0	0	0	0	0	0	0	0
Hydroxyproline	1258	1383	1404	1396	1415	1477	1478	1274	1472	1784	1648	1082	1003	1057	335	285	346
Creatine	53629	53510	62565	58524	58634	64210	60852	60997	65183	65314	70206	72649	75560	74936	38378	36489	37045
Ile	51749	55923	60510	53716	58428	58827	61845	58737	60860	90501	75905	39421	40356	40678	13713	15219	13126
Leu	53066	57972	63263	56116	58301	62458	65360	61414	62016	93531	79350	41214	41856	42046	14252	15756	14009
Asn	2100	2179	2518	2096	2398	2664	3185	3791	3682	5502	5738	5265	5665	5503	1437	1455	1553
Gly-Gly	131	254	210	160	195	185	108	161	200	114	224	124	86	0	0	0	0
Ornithine	645	743	688	637	718	634	1469	1175	975	3192	4855	1960	2186	2172	1269	1286	1443
Asp	68801	79816	87039	45246	50186	50643	41967	45746	48226	49612	50244	45336	52371	46515	6443	5624	7609
Adenine	100	138	132	99	175	143	194	158	185	369	340	246	315	300	253	300	247
Hypoxanthine	188	276	310	0	0	213	301	0	0	676	445	179	257	151	365	292	395
gamma-Guanidinobutyrate	0	0	0	0	0	0	0	0	0	0	12	0	0	0	0	0	0
gamma-Butyrobetaine	108	113	120	107	119	120	113	102	104	125	116	102	101	97	43	43	44
Gln	242409	262259	286013	256262	271526	280285	281939	273625	281612	353128	309939	190058	190580	197256	58515	59664	58446
Lys	10355	14254	11179	9927	10644	10265	18555	19039	15403	77632	53591	31454	36392	33775	23387	26962	24374
Glu	314351	354960	378813	326746	368733	383113	362843	390234	406044	412841	434184	359614	377957	373663	183960	179159	191070
Met	10908	11798	13208	11284	12075	12861	12688	11936	12824	18282	14158	7992	7180	7915	2580	2430	2540
His	14469	15848	16422	12742	15664	14633	16412	17052	16683	23776	21292	10393	11162	11061	3530	4146	3743
alpha-Aminoadipate	378	379	433	331	406	329	378	446	443	350	384	330	380	379	128	127	153
Carnitine	362	378	432	421	477	475	492	465	482	497	556	440	432	421	209	203	196
Methionine sulfoxide	109	181	148	108	211	172	164	178	132	244	341	155	177	183	146	130	119
Phe	32755	37415	39100	33304	37216	37749	37985	36937	38237	54964	46242	22299	23675	24056	7675	8796	7773
Pyridoxal	37	57	0	36	0	31	39	28	0	40	29	45	56	51	50	66	66
Pyridoxine	101	144	144	99	110	126	170	144	126	525	310	94	131	133	92	125	103
3-Methylhistidine	151	153	177	142	155	167	191	178	188	268	220	114	132	109	48	63	54
Arg	2930	3630	3236	2916	3008	2825	6603	5336	4188	26777	15251	8475	9777	9372	6100	6889	6321
Citrulline	7817	8801	8959	7772	8747	9233	8147	8295	8553	8292	9195	7041	7251	6937	5635	5896	6527
Tyr	27278	31732	33989	27124	31717	33013	32085	31821	32071	44653	40487	19162	20662	20624	6380	7482	6669
Phosphorylcholine	33466	36902	40172	33154	36934	36986	32836	33277	33889	30027	31666	23902	25028	24323	11259	10638	11883
SAH	57	0	0	0	0	0	0	0	0	0	0	0	0	0	0	0	0
SAM+	1119	1009	1118	1188	1197	1202	1652	1860	1792	2674	2259	2923	2880	2805	2004	1955	2270
o-Acetylcarnitine	88	102	113	70	94	97	73	72	66	44	45	49	55	54	20	20	16
Trp	6447	7093	7811	7252	7355	7749	8031	7489	7736	10934	9111	4960	5217	5254	1743	1900	1726
Cystathionine	999	1287	1201	1159	1080	1176	1094	1153	1191	1081	1091	827	942	931	479	497	438
Melatonin	68	106	0	85	66	0	96	0	0	0	0	0	0	0	93	0	0
Cytidine	76	121	94	110	59	84	77	137	159	146	143	80	89	89	70	85	66
Pyridoxamine 5'-phosphate	35	0	0	0	72	0	0	0	47	0	51	0	33	0	0	0	35
Glycerophosphorylcholine	5103	6123	6860	4902	5307	5415	4598	3590	3729	3068	4407	3786	3372	4086	1916	2223	1868
Thiamine	323	378	313	279	318	209	404	416	371	1044	650	272	305	291	273	319	228
Adenosine	48	58	54	57	0	58	90	51	70	52	37	0	32	0	81	66	87
Inosine	195	247	0	226	147	232	296	262	162	142	151	227	206	224	747	645	712
Glu-Glu	0	44	25	25	0	0	23	33	31	35	37	0	12	21	0	0	0
Guanosine	155	180	141	200	114	144	129	182	201	0	0	69	113	64	249	317	352
Ophthalmate	0	130	82	60	82	0	83	97	98	70	82	141	107	118	85	0	93
5-Methylthioadenosine	29	27	27	27	36	30	34	46	41	57	75	48	66	68	45	54	38
Glutathione(ox)	23616	24580	11180	9530	13270	8318	14335	14566	15288	20535	18183	13327	13822	16232	13020	13134	14721
Glutathione(red)	30593	29731	58281	52789	48139	56917	42621	41580	43551	26882	36883	36590	36976	33828	6377	5691	1345
Saccharopine	63	61	61	57	72	65	67	84	71	70	71	67	75	47	46	51	0
Pyruvate	0	0	0	0	0	0	0	0	0	11673	5199	0	0	0	0	0	0
Lactate	25462	29149	26368	27192	33440	33815	35872	35314	34107	86511	59823	61461	66242	63929	74842	74817	72542
3-Hydroxybutyrate	0	233	152	0	0	0	0	0	139	301	157	0	0	0	0	0	0
Fumarate	1088	1633	1253	964	1195	1050	907	1078	1291	1045	1033	1352	1863	1675	1012	1173	1323
2-Oxoisopentanoate	0	181	137	0	164	146	190	124	116	379	166	0	0	0	0	0	0
4-Oxopentanoate	0	151	95	0	0	118	0	95	0	0	64	0	0	0	0	0	135
Hexanoate	107	111	68	102	77	91	86	65	94	91	96	86	120	95	139	108	88
Succinate	635	940	530	399	549	403	498	400	453	1230	752	230	292	268	411	569	441
2-Hydroxypentanoate	156	180	232	105	221	134	139	181	140	189	151	143	175	99	148	167	115
5-Oxoproline	5042	6547	4867	4901	5256	4679	7815	5896	5760	24191	14169	7260	7548	7392	7470	8591	7004
4-Methyl-2-oxopentanoate	663	905	737	587	581	598	715	611	566	1978	1086	314	458	339	355	467	320
N-Acetyl-beta-alanine	252	290	250	385	324	278	356	238	344	232	256	223	328	220	139	0	114
Glutarate	0	0	135	0	162	183	191	189	275	324	177	0	289	212	0	179	0
Malate	4929	5636	4873	3695	5027	3795	3168	3442	3722	3323	3221	4724	5934	4916	3921	4292	4259
Ethanolamine phosphate	101	97	76	98	80	85	97	57	104	279	223	870	851	995	984	889	954
2-Oxoglutarate	704	701	922	773	472	528	613	484	700	587	370	0	306	0	579	464	443
2-Hydroxyglutarate	0	223	170	162	263	139	223	144	230	214	216	163	307	231	0	239	173
trans-Cinnamate	4212	3982	4355	4152	3959	4262	4490	4434	4736	4607	4651	4143	4451	4269	2216	2319	2213
Pelargonate	71	80	70	100	89	78	89	56	51	78	53	59	51	58	70	64	77
3-Hydroxy-3-methylglutarate	0	0	0	0	110	0	0	57	76	0	78	0	0	0	0	0	0
Terephthalate	56	76	69	0	63	0	0	46	64	39	47	27	62	59	0	0	60
PEP	85	60	42	0	53	0	59	40	42	58	62	71	95	37	41	0	37
Cysteate	0	0	27	0	0	21	35	21	31	34	34	17	0	0	0	0	0
DHAP	4637	4574	4163	4485	4541	4054	4705	4159	5639	3363	4278	4161	3975	4043	5360	5239	5898
Glycerophosphate	330	356	287	339	246	310	273	235	354	369	433	299	305	325	3071	3207	3519
Decanoate	43	66	47	46	37	57	55	50	50	0	33	45	37	37	61	66	0
cis-Aconitate	98	113	115	0	49	0	0	0	0	0	0	0	0	0	0	0	0
N-Acetylaspartate	644	742	564	566	783	607	566	558	632	556	616	448	595	562	277	319	309
Carbamoylaspartate	119	183	182	0	96	111	145	0	90	0	0	0	0	0	0	0	0
O-Phosphoserine	143	161	176	71	0	124	95	50	0	119	0	0	0	0	0	0	0
3PG	246	260	197	131	211	190	205	259	213	323	220	218	192	205	171	178	148
Azelate	46	36	25	33	30	31	55	19	20	20	15	36	22	29	38	20	41
N-Acetylglutamate	35	38	31	39	46	38	66	56	45	57	49	63	52	45	0	0	26
N-Acetylmethionine	73	108	89	103	107	57	81	97	79	0	69	0	44	56	0	0	0
Isocitrate	0	50	46	0	48	17	31	0	39	60	0	0	0	0	0	0	0
Citrate	2161	2265	2176	873	957	682	513	380	448	755	336	162	141	160	262	230	179
Dodecanoate	145	85	117	121	122	101	113	104	120	104	145	113	129	103	141	144	136
Mucate	0	0	0	0	0	0	0	0	0	0	0	18	0	0	0	0	0
Pantothenate	544	634	551	562	638	522	617	602	621	916	692	477	448	430	194	206	233
R5P	477	485	366	479	373	452	567	456	516	395	517	612	549	660	680	642	716
Ru5P	1258	1388	1365	1509	1347	1472	1656	1557	1456	1498	1455	1655	1674	1734	1955	1702	1875
Biotin	1445	1434	1737	1322	1333	1646	1457	1367	1481	1308	1195	1078	1023	1056	516	483	572
Glucosamine 6-phosphate	0	0	0	0	34	0	0	30	0	0	24	0	0	0	0	0	0
G6P	477	572	647	417	340	307	707	534	536	851	737	695	753	719	529	470	539
F6P	166	154	171	127	79	118	187	150	122	175	130	205	220	183	203	172	190
G1P	318	284	315	448	444	455	542	570	608	669	611	590	674	677	949	945	1011
Sorbitol 6-phosphate	0	0	0	0	0	0	0	0	0	0	0	0	0	0	0	0	0
2,3-DPG	65	60	74	0	51	75	64	142	59	64	69	36	29	20	0	0	31
6-Phosphogluconate	975	946	856	505	502	527	796	825	692	872	844	756	819	750	519	457	540
S7P	97	124	121	171	138	103	55	133	144	163	149	119	174	156	93	114	128
Argininosuccinate	137	148	142	125	124	92	138	146	171	170	150	280	344	333	161	170	198
N-Acetylglucosamine 1-phosphate	52	41	47	49	43	33	42	31	45	44	44	46	30	43	60	57	54
dCMP	0	0	0	0	0	0	0	0	0	0	0	0	0	0	0	0	0
N-Acetylneuraminate	78	64	37	46	41	43	77	73	69	269	110	120	126	109	169	135	169
Ribulose 1,5-diphosphate	55	76	75	71	77	57	97	119	115	217	247	339	355	314	428	390	387
dTMP	0	43	29	28	0	19	0	16	27	0	14	0	0	0	0	0	0
CMP	138	168	165	190	79	107	226	118	146	85	55	69	59	60	76	54	60
UMP	707	753	648	631	321	362	650	431	476	485	266	158	129	141	212	208	228
dAMP	0	0	0	0	0	28	0	0	0	0	0	0	0	0	0	0	0
F1,6P	6466	6828	6360	5054	4444	4254	7455	9422	8091	7954	8590	8816	9688	9330	12612	11695	13817
AMP	1251	1407	1273	1514	546	710	1621	959	1106	446	279	237	297	225	346	365	331
IMP	148	139	128	143	82	109	164	130	94	102	105	185	148	134	395	453	412
GMP	190	170	221	205	121	134	253	149	181	163	124	107	109	108	216	257	242
XMP	0	0	0	0	0	0	0	0	0	0	0	0	0	0	0	0	9.5
NADPH	0	0	174	0	148	142	0	191	0	188	218	129	106	117	0	0	131
CoA	0	56	75	0	0	45	0	116	81	0	128	87	128	113	0	0	0
dCDP	0	0	0	0	0	0	0	9.3	0	0	0	0	0	0	0	0	0
PRPP	0	0	0	0	0	0	0	0	0	105	0	0	120	0	0	110	0
dTDP	26	0	0	0	0	0	37	22	20	0	0	0	0	0	0	0	0
CDP	326	278	266	215	148	159	347	340	299	241	179	98	91	119	89	82	75
UDP	749	853	722	542	369	331	891	836	665	601	390	214	192	172	171	101	159
dADP	0	0	0	0	0	0	0	15	0	0	14	0	0	0	0	12	0
Trehalose 6-phosphate	0	0	0	0	0	0	0	0	0	0	0	0	0	0	0	0	0
Adenosine 5'-phosphosulfate	0	0	0	0	0	0	0	0	0	0	0	0	0	0	0	0	0
ADP	3179	2918	2719	2193	1492	1292	3189	3262	2883	2269	1770	1131	1151	1156	1014	914	968
GDP	661	525	537	450	326	320	824	800	706	485	379	301	329	300	504	428	474
Adenylosuccinate	0	0	29	0	0	0	0	0	0	0	0	0	0	0	0	0	0
dCTP	34	35	33	0	38	0	0	27	29	26	34	42	40	35	0	0	0
dTTP	62	45	52	30	45	31	31	70	46	96	59	92	52	72	0	0	39
CTP	857	674	727	475	598	528	707	1113	855	1346	1524	1594	1465	1491	450	352	469
UTP	2163	1801	1728	1014	1707	1320	1641	2703	1913	2994	3116	2649	2310	2163	607	503	582
dATP	67	0	46	0	0	36	40	53	56	39	40	87	90	61	0	0	62
ATP	8469	6217	7008	3611	5787	5267	5617	9888	7681	10106	11263	9976	9146	9073	5554	4663	5325
ITP	817	592	663	367	555	485	563	949	779	922	1046	894	848	882	590	480	541
GTP	2765	1997	1875	1369	1997	2044	2772	4237	2810	2948	3614	4491	3588	3807	3014	2612	2913
CDP-choline	85	79	94	145	98	105	126	145	164	160	212	1309	962	1255	3581	2828	3267
UDP-glucose	1323	1109	1027	841	916	685	1155	1172	1087	1217	1119	832	734	663	523	413	473
UDP-glucuronate	854	482	482	357	431	375	610	1182	630	1530	1427	958	899	865	744	616	715
ADP-glucose	48	39	46	40	45	42	49	57	46	102	71	74	53	82	63	44	52
GDP-mannose	67	53	50	55	68	53	75	99	72	142	150	163	140	164	163	125	162
UDP-N-acetylglucosamine	1547	1293	1232	856	904	748	1253	1253	1146	1727	1488	1328	1255	1176	951	796	913
CMP-N-acetylneuraminate	142	83	107	137	106	118	169	173	144	303	225	240	205	189	145	146	162
NAD+	1519	1304	1428	1422	1238	1113	1429	1401	1375	1357	1292	1188	1200	1182	774	649	739
NADH	[illegible]	[illegible]	[illegible]	[illegible]	[illegible]	[illegible]	[illegible]	[illegible]	[illegible]	[illegible]	[illegible]	[illegible]	[illegible]	[illegible]	[illegible]	[illegible]	[illegible]
NADP+	[illegible]	[illegible]	[illegible]	[illegible]	[illegible]	[illegible]	[illegible]	[illegible]	[illegible]	[illegible]	[illegible]	[illegible]	[illegible]	[illegible]	[illegible]	[illegible]	[illegible]
FAD	[illegible]	[illegible]	[illegible]	[illegible]	[illegible]	[illegible]	[illegible]	[illegible]	[illegible]	[illegible]	[illegible]	[illegible]	[illegible]	[illegible]	[illegible]	[illegible]	[illegible]
Glu+ threo-beta-methylaspartate	[illegible]	[illegible]	[illegible]	[illegible]	[illegible]	[illegible]	[illegible]	[illegible]	[illegible]	[illegible]	[illegible]	[illegible]	[illegible]	[illegible]	[illegible]	[illegible]	[illegible]
Threonate	[illegible]	[illegible]	[illegible]	[illegible]	[illegible]	[illegible]	[illegible]	[illegible]	[illegible]	[illegible]	[illegible]	[illegible]	[illegible]	[illegible]	[illegible]	[illegible]	[illegible]
G3P	[illegible]	[illegible]	[illegible]	[illegible]	[illegible]	[illegible]	[illegible]	[illegible]	[illegible]	[illegible]	[illegible]	[illegible]	[illegible]	[illegible]	[illegible]	[illegible]	[illegible]
2PG	[illegible]	[illegible]	[illegible]	[illegible]	[illegible]	[illegible]	[illegible]	[illegible]	[illegible]	[illegible]	[illegible]	[illegible]	[illegible]	[illegible]	[illegible]	[illegible]	[illegible]

Table S2. Metabolic changes induced by SBA, BA in HSC-2 cells (Exp. I), and by eugenol in HGF, HPC and HPLF cells (Exp. II). 188 compounds were detected. Each value represents amol/cell, and can be seen by magnifier.

#NAME?	control			4mM SBA			8mM SBA			8mM benzaldehyde			[illegible]
	1a	2a	3a	4a	5a	6a	7a	8a	9a	10a	11a	12a	[illegible]
Gly	12923	11681	13879	15742	15071	16640	17023	16742	17652	16012	16765	15824	[illegible]
Putrescine(1,4-Butanediamine)	45	33	63	40	35	47	33	50	41	27	31	37	[illegible]
Sarcosine	0	0	39	38	31	48	40	28	31	46	54	0	[illegible]
beta-Ala	914	737	866	873	888	919	911	909	926	267	365	290	[illegible]
Ala	6409	5773	6572	7045	6656	7153	7277	7093	7144	4730	4938	4506	[illegible]
N,N-Dimethylglycine	0	0	29	0	11	24	0	0	0	21	0	0	[illegible]
2-Aminobutyrate;2AB	129	118	132	149	145	144	154	151	157	122	132	122	[illegible]
GABA	1331	1201	1335	1301	1323	1504	1367	1377	1432	205	303	236	[illegible]
Choline	136	138	195	171	193	194	154	156	211	450	433	464	[illegible]
Ser	9982	8963	10245	10734	10375	10906	11145	11048	11584	9056	9692	8820	[illegible]
Diethanolamine	82	69	40	92	83	94	66	74	104	62	38	45	[illegible]
Hypotaurine	1534	1349	1665	1553	1623	1734	1654	1681	1730	1446	1622	1533	[illegible]
Carnosine divalent;Carnosine	8.6	8.0	8.2	10	4.7	10	7.9	8.3	9.3	7.8	9.3	11	[illegible]
Creatinine	164	145	202	177	171	182	166	174	176	176	177	188	[illegible]
Pro	8002	7224	8324	6481	6320	7050	6340	6254	6447	15087	14960	14603	[illegible]
Val	9912	8559	10191	10527	9928	10795	10931	10502	11076	10495	11655	10692	[illegible]
Betaine	194	176	171	171	190	186	182	168	199	209	199	222	[illegible]
5-Aminovalerate	299	265	264	299	289	289	297	296	281	71	63	65	[illegible]
Thr	13757	12418	14938	14808	14298	15681	15529	15394	16216	12881	14262	13180	[illegible]
Homoserine	0	0	17	36	0	0	21	0	33	39	20	25	[illegible]
Nicotinamide	72	58	103	90	53	87	81	72	73	97	94	114	[illegible]
Hydroxyproline	272	258	280	300	265	320	310	307	314	253	281	260	[illegible]
Creatine	9970	8710	10337	15663	14508	15597	17153	16468	17150	18869	16369	17300	[illegible]
Ile	9398	8459	9890	10024	9440	10765	10506	10189	10887	10562	11685	10811	[illegible]
Leu	9867	8814	10605	10550	10002	11000	10773	10728	11301	10826	12093	11389	[illegible]
Asn	399	347	397	418	361	381	444	425	438	1172	1170	1139	[illegible]
Gly-Gly	69	62	82	76	63	79	78	72	72	56	57	46	[illegible]
Ornithine	112	115	114	125	122	127	116	102	123	506	463	516	[illegible]
Asp	14482	12369	14124	17245	15590	16349	16499	15257	16504	10475	10027	9500	[illegible]
Hypoxanthine	43	0	43	22	0	0	0	0	0	30	0	0	[illegible]
1-Methylnicotinamide	0	0	0	0	0	0	0	0	0	0	0	0	[illegible]
Urocanate	0	28	0	0	0	0	0	0	0	0	0	0	[illegible]
Hexamine	0	0	0	0	0	0	0	0	0	0	0	0	[illegible]
gamma-Guanidinobutyrate	3.8	3.4	2.8	0	3.7	3.5	3.7	3.9	3.8	4.6	5.3	2.5	[illegible]
gamma-Butyrobetaine	75	66	83	70	67	80	70	73	75	73	77	71	[illegible]
Acetylcholine	3.4	1.3	0	0	0	0	0	0	0	0	0	0	[illegible]
Spermidine	11	8.9	15	11	7.1	12	6.6	9.5	10	16	14	18	[illegible]
Gln	53140	46706	53273	55885	54416	58952	60385	58578	61799	53708	57270	53047	[illegible]
Lys	2563	1960	2915	2906	2384	2897	2907	2716	2773	8823	8269	8770	[illegible]
Glu	86322	75253	83899	91059	84174	92298	87161	81421	85473	69632	69747	67005	[illegible]
Met	2156	1736	2463	2254	2365	2532	2314	2585	2519	2092	2636	2302	[illegible]
Triethanolamine	23	26	17	20	19	21	20	12	24	24	14	22	[illegible]
His	2574	2267	2643	2726	2633	2775	2814	2704	2878	2525	2844	2590	[illegible]
alpha-Aminoadipate	220	180	230	254	191	237	198	185	188	267	230	230	[illegible]
Carnitine	218	208	242	212	205	226	215	220	220	325	310	314	[illegible]
5-Hydroxylysine	9.5	5.1	8.9	0	0	0	0	7.2	0	8.6	0	8.2	[illegible]
Methionine sulfoxide	72	81	52	76	51	60	66	59	59	88	58	53	[illegible]
Phe	5706	4991	6087	6015	5610	6388	6336	6149	6361	5792	6287	5873	[illegible]
Pyridoxal	0	156	159	0	169	147	178	141	182	138	176	197	[illegible]
Pyridoxine	60	52	82	66	63	69	65	59	66	56	69	73	[illegible]
3-Methylhistidine	74	63	89	87	81	84	81	81	92	77	92	79	[illegible]
Arg	695	494	764	829	666	792	809	752	761	2338	2204	2426	[illegible]
Citrulline	940	803	956	980	868	1030	999	924	1002	1133	1172	1140	[illegible]
Glucosamine	0	0	7.9	9.5	0	10	8.5	11	0	9.7	16	0	[illegible]
Tyr	5898	5105	6044	6180	5806	6429	6572	6253	6441	6082	6554	5942	[illegible]
Phosphorylcholine	15765	14051	14862	16035	15648	16708	15535	15705	15610	11548	12067	11099	[illegible]
Gly-Leu	0	0	8.8	0	0	8.9	0	8.1	11	0	0	0	[illegible]
N6,N6,N6-Trimethyllysine	8.8	7.3	8.4	12	10	8.0	8.3	8.2	11	11	14	0	[illegible]
S-Adenosylhomocysteine divalent;SAH	18	17	20	19	19	22	19	14	14	21	21	20	[illegible]
S-Adenosylmethionine divalent;SAM+	196	144	172	215	178	187	184	169	173	357	324	296	[illegible]
o-Acetylcarnitine	80	73	87	117	109	125	114	120	119	75	66	74	[illegible]
Trp	1165	994	1175	1361	1215	1380	1445	1360	1412	1314	1388	1282	[illegible]
Kynurenine	0	0	6.5	8.3	9.5	10	12	7.0	0	8.2	0	0	[illegible]
Cysteine-glutathione disulphide -Divalent	8.1	11	0	18	0	0	109	26	0	33	0	0	[illegible]
Cystathionine	233	219	225	240	236	248	224	237	242	229	235	245	[illegible]
Cytidine	28	16	24	0	18	21	21	19	17	28	26	23	[illegible]
Pyridoxamine 5'-phosphate	0	0	0	21	21	0	0	0	0	20	25	25	[illegible]
Glycerophosphorylcholine	1752	1464	1547	1971	1741	2016	2005	1914	1997	1624	1367	1363	[illegible]
Thiamine	12442	10347	13968	12363	10703	13300	12579	11260	11969	14420	15470	15442	[illegible]
Adenosine	14	4.6	13	6.8	0	7.2	6.1	5.0	5.6	14	10	15	[illegible]
Inosine	53	0	0	0	0	0	0	0	0	78	52	80	[illegible]
Glu-Glu	13	12	13	15	17	18	15	14	19	13	12	10	[illegible]
Guanosine	39	12	28	9.5	0	0	0	0	0	26	23	30	[illegible]
Ophthalmate	19	13	14	22	19	23	33	23	18	38	34	39	[illegible]
5-Methylthioadenosine	22	18	35	19	22	26	23	26	23	45	49	47	[illegible]
Glutathione(ox) divalent;Glutathione(ox)	2030	1913	2410	2829	2601	3812	6950	4804	3950	2575	2317	3045	[illegible]
Glutathione(red)	32844	30512	29738	32518	29969	28557	18781	23392	27182	27036	27377	23405	[illegible]
Thiamine monophosphate	2041	1367	1490	2228	1927	0	1659	0	1539	0	0	0	[illegible]
S-Lactoylglutathione	30	30	30	28	20	29	27	24	33	29	35	30	[illegible]
Glycolate	357	314	367	322	375	341	330	0	366	0	564	0	[illegible]
Pyruvate	908	1036	1451	1042	992	1437	964	1182	1180	819	964	1069	[illegible]
Butanoate	0	0	0	0	1745	0	1016	0	0	0	0	0	[illegible]
Lactate	12576	17048	17311	13973	15702	17106	15137	15152	15565	30189	28697	32033	[illegible]
3-Methylbutanoate	0	0	0	0	0	0	0	0	0	0	0	0	[illegible]
Malonate	0	388	0	0	0	0	0	0	0	776	426	610	[illegible]
3-Hydroxybutyrate	55	45	69	62	44	51	0	57	65	63	0	75	[illegible]
2-Hydroxyisobutyrate	0	0	16	0	0	0	0	0	0	0	0	0	[illegible]
Fumarate	535	614	648	574	522	554	538	455	517	661	595	591	[illegible]
2-Oxoisopentanoate	69	85	115	71	68	85	90	86	63	42	39	58	[illegible]
4-Oxopentanoate	71	84	102	48	60	107	78	92	88	80	63	71	[illegible]
Hexanoate	18	33	37	24	31	36	39	36	31	25	42	33	[illegible]
Succinate	146	111	170	214	184	211	213	178	185	87	109	100	[illegible]
2-Hydroxypentanoate	89	92	147	96	74	94	115	86	94	93	73	82	[illegible]
Benzoate	226	0	327	0	0	0	469	339	284	314	905	613	[illegible]
Isethionate	36	0	123	0	59	0	0	0	0	0	0	94	[illegible]
5-Oxoproline	193412	177254	261310	199368	179962	213751	201051	189400	172531	390124	357721	399161	[illegible]
Citraconate	1312	0	859	829	0	868	1260	1240	0	1331	0	0	[illegible]
4-Methyl-2-oxopentanoate	367	298	440	379	329	375	358	336	343	222	168	227	[illegible]
4-Acetylbutyrate	18	25	24	20	27	20	23	27	28	28	19	32	[illegible]
N-Acetyl-beta-alanine	155	154	191	147	136	156	141	153	147	181	193	153	[illegible]
Glutarate	78	73	101	90	52	87	69	61	73	81	82	84	[illegible]
Malate	1799	1884	2145	1945	1772	1947	2101	1839	1898	2293	1835	1982	[illegible]
o-Hydroxybenzoate	35	34	49	32	30	33	44	30	34	44	43	41	[illegible]
Phosphonoacetate	0	0	0	1355	1518	1695	1414	2057	1907	0	0	0	[illegible]
Carbamoylphosphate	1179	2634	2509	2080	2260	2467	2421	2910	2656	3063	2873	2792	[illegible]
Ethanolamine phosphate	26	38	36	27	32	29	33	33	36	162	98	150	[illegible]
Octanoate	0	0	40	0	0	0	46	31	32	0	0	0	[illegible]
2-Oxoglutarate	434	0	618	539	0	526	519	0	668	0	0	0	[illegible]
Adipate	0	0	40	0	0	0	0	0	0	0	0	0	[illegible]
threo-beta-methylaspartate	49658	50354	54772	53293	53995	56819	54529	51341	54144	42476	41954	41026	[illegible]
2-Hydroxyglutarate	60	66	71	83	67	56	72	73	67	87	98	87	[illegible]
trans-Cinnamate	1355	1309	1510	1492	1481	1479	1429	1408	1471	1060	1154	1107	[illegible]
3-Phenylpropionate	285	226	406	99	104	281	386	287	352	183	335	362	[illegible]
Pelargonate	38	38	74	40	54	34	75	79	71	59	75	75	[illegible]
3-Hydroxy-3-methylglutarate	26	29	53	32	43	38	49	40	34	31	25	28	[illegible]
PEP	54	61	55	307	349	326	261	287	378	37	37	34	[illegible]
Cysteate	418	0	278	309	0	0	0	0	0	0	0	0	[illegible]
DHAP	1608	1347	1652	1222	1184	1440	1538	1382	1404	1611	1796	1702	[illegible]
Glycerophosphate	218	230	260	237	243	278	295	270	271	279	293	269	[illegible]
Decanoate	42	24	52	33	34	0	46	60	49	0	45	60	[illegible]
N-Acetylleucine	4.3	0	3.5	3.3	1.7	5.4	0	3.7	0	5.3	0	0	[illegible]
cis-Aconitate	34	37	40	50	39	50	64	61	51	0	11	8.8	[illegible]
N-Acetylaspartate	613	610	713	628	552	629	645	592	599	742	642	646	[illegible]
Carbamoylaspartate	84	86	86	62	58	73	63	49	0	0	42	0	[illegible]
O-Phosphoserine	147	120	159	293	226	322	367	359	352	11	16	12	[illegible]
3PG	269	221	238	728	777	758	582	640	860	166	155	154	[illegible]
Undecanoate	10791	0	12946	0	0	0	14531	15601	23328	12225	11849	19694	[illegible]
Azelate	21	15	23	16	12	30	13	16	16	22	13	13	[illegible]
N-Acetylglutamate	46	41	50	47	37	45	36	44	45	47	47	43	[illegible]
N-Acetylmethionine	26	25	28	30	26	19	19	21	22	33	26	25	[illegible]
Citrate	884	820	889	1355	1096	1191	1378	1259	1305	131	100	106	[illegible]
Syringate	1700	1982	0	0	2102	1923	2274	0	2248	1543	2431	2911	[illegible]
Dodecanoate	65	0	83	45	54	51	97	79	85	63	77	99	[illegible]
Pantothenate	308	324	345	272	283	298	293	292	270	286	307	286	[illegible]
Sinapate	0	0	0	279	203	231	610	374	356	0	0	0	[illegible]
R5P	62	68	110	74	95	90	75	73	91	117	136	180	[illegible]
Ru5P	334	306	288	396	344	451	413	395	416	402	450	453	[illegible]
Biotin	537	578	575	568	573	625	586	596	638	415	442	451	[illegible]
G6P	230	256	327	456	493	500	471	477	461	318	336	305	[illegible]
F6P	0	0	0	0	0	145	0	0	0	111	0	106	[illegible]
G1P	138	0	0	143	114	0	139	0	133	0	470	447	[illegible]
2,3-DPG	280	274	242	322	281	334	290	288	320	89	107	92	[illegible]
6-Phosphogluconate	830	794	818	4713	4397	4946	4656	4489	4607	550	507	511	[illegible]
S7P	113	114	114	112	116	122	144	134	131	226	205	192	[illegible]
Argininosuccinate	32	39	29	38	35	46	51	37	32	129	89	109	[illegible]
N-Acetylglucosamine 1-phosphate	34	33	37	30	31	33	46	52	38	29	20	27	[illegible]
N-Acetylneuraminate	73	49	71	42	53	78	88	75	61	104	122	110	[illegible]
Ribulose 1,5-diphosphate	240	154	224	269	235	275	308	305	332	842	807	801	[illegible]
CMP	155	48	118	42	25	40	65	31	29	83	70	75	[illegible]
UMP	178517	84230	145456	87346	50524	79349	86029	67128	63486	67761	57328	73132	[illegible]
F1,6P	5779	3350	4887	3438	2506	3222	3909	3085	3258	8137	7541	8127	[illegible]
AMP	1129	282	825	304	113	208	347	216	179	436	321	388	[illegible]
AMP	1129	270	825	304	113	208	347	216	179	436	321	388	[illegible]
IMP	61	28	32	37	15	23	25	27	25	135	66	92	[illegible]
GMP	189	62	126	81	48	61	84	59	53	99	79	89	[illegible]
XMP	0	0	0	0	0	0	0	0	8.1	0	0	0	[illegible]
NADPH	345	298	209	288	222	291	240	210	327	128	149	122	[illegible]
CoA	64	61	73	69	66	73	63	55	57	92	82	82	[illegible]
PRPP	129	61	50	143	58	87	131	72	96	534	286	340	[illegible]
dTDP	20	0	21	11	0	13	14	0	11	19	19	20	[illegible]
CDP	298	119	220	129	65	99	143	113	108	216	194	225	[illegible]
UDP	21112	8987	18421	10370	6072	7723	11611	8523	7806	11213	10981	11354	[illegible]
Acetyl CoA	0	0	0	0	0	0	0	0	0	0	0	0	[illegible]
dADP	2396	0	1220	1124	0	0	1117	823	0	1322	1296	1746	[illegible]
Trehalose 6-phosphate	0	0	0	7141	0	5243	10364	8658	6322	0	0	0	[illegible]
Adenosine 3',5'-diphosphate	703	828	0	0	0	0	0	772	0	949	0	0	[illegible]
ADP	2337	1021	1812	1267	805	1078	1420	1084	1023	1528	1308	1403	[illegible]
GDP	524	171	375	235	128	204	270	186	174	319	273	279	[illegible]
Adenylosuccinate	28	0	29	13	0	0	15	0	0	27	16	23	[illegible]
dCTP	25	24	23	26	26	25	23	37	22	21	22	17	[illegible]
dTTP	46	51	45	63	57	65	59	63	66	58	48	53	[illegible]
CTP	584	685	487	846	775	804	846	769	860	817	793	740	[illegible]
UTP	1689	2105	1623	2328	2375	2431	2356	2279	2445	1720	1807	1690	[illegible]
dATP	29	33	24	33	32	35	37	39	22	33	27	31	[illegible]
[illegible]	[illegible]	[illegible]	[illegible]	[illegible]	[illegible]	[illegible]	[illegible]	[illegible]	[illegible]	[illegible]	[illegible]	[illegible]	[illegible]
